# Advances and Prospects of Phenolic Acids Production, Biorefinery and Analysis

**DOI:** 10.3390/biom10060874

**Published:** 2020-06-06

**Authors:** Egle Valanciene, Ilona Jonuskiene, Michail Syrpas, Ernesta Augustiniene, Paulius Matulis, Andrius Simonavicius, Naglis Malys

**Affiliations:** Bioprocess Research Centre, Faculty of Chemical Technology, Kaunas University of Technology, Radvilėnų pl. 19, Kaunas LT-50254, Lithuania; egle.valanciene@ktu.lt (E.V.); ilona.jonuskiene@ktu.lt (I.J.); michail.syrpas@ktu.lt (M.S.); ernesta.augustiniene@ktu.lt (E.A.); paulius.matulis@ktu.lt (P.M.); andrius.simonavicius@ktu.lt (A.S.)

**Keywords:** phenolic acid, antioxidant activity, biorefinery, extraction, analytical methods, metabolic engineering, microbial fermentation, biosensor

## Abstract

Biotechnological production of phenolic acids is attracting increased interest due to their superior antioxidant activity, as well as other antimicrobial, dietary, and health benefits. As secondary metabolites, primarily found in plants and fungi, they are effective free radical scavengers due to the phenolic group available in their structure. Therefore, phenolic acids are widely utilised by pharmaceutical, food, cosmetic, and chemical industries. A demand for phenolic acids is mostly satisfied by utilising chemically synthesised compounds, with only a low quantity obtained from natural sources. As an alternative to chemical synthesis, environmentally friendly bio-based technologies are necessary for development in large-scale production. One of the most promising sustainable technologies is the utilisation of microbial cell factories for biosynthesis of phenolic acids. In this paper, we perform a systematic comparison of the best known natural sources of phenolic acids. The advances and prospects in the development of microbial cell factories for biosynthesis of these bioactive compounds are discussed in more detail. A special consideration is given to the modern production methods and analytics of phenolic acids.

## 1. Introduction

Phenolic acids, alongside simple phenols, hydrolysable tannins, acetophenones, lignans, coumarins, benzophenones, xanthones, stilbenes, and secoiridoids, belong to a large group of aromatic compounds, often referred to as phenolic compounds or phenolics [1,2,3]. Phenolics are secondary metabolites that are naturally biosynthesised by plants and fungi. They contribute to diverse biological functions ranging from signalling and structural to defensive against biotic (infection, limitation, or excess of nutrients) and abiotic (extreme temperature, UV, and visible light) factors [4,5,6]. Phenolics have been proven to offer multiple health benefits to humans and animals. For their antioxidative, anticarcinogenic, anti-inflammatory, and antibacterial activities involving free radical scavenging, metal chelation, reducing capacity, and contribution to the modulation of signal transduction and enzymatic activity, phenolics are implicated in reducing the risk of cancer, as well as infectious and cardiovascular diseases [7,8]. Moreover, phenolics provide different qualities to food and beverages including colour, flavour, bitterness, odour, and preservative characteristics [9].

Phenolic acids form one of the largest groups of phenolic compounds and are distinguishable from other phenolics by their structure, which typically contains a carboxyl group and one or more hydroxyl groups bonded to the aromatic ring [10]. The hydroxyl groups and other functional groups attached to the aromatic ring determine the bioactive properties of phenolic acids [11]. Similarly to other phenolic compounds, the phenolic acids exhibit antioxidant, antimicrobial, antiviral, antimutagenic, or anticancer activity [12,13,14,15,16,17]. They reduce oxidative damage or stress in the cells, reducing the risk of diseases. Phenolic acids such as syringic (**11**), gentisic (**12**), and gallic (**8**) acids show activity in chronic live injuries, diabetes, memory loss, and wound healing [18,19,20,21,22]. Anti-aging, anti-wrinkle, and skin whitening effects have been for ferulic and gallic acids [23,24]. Other promising therapeutic applications of phenolic acids have recently been reviewed in [1].

Importantly, phenolic acids are industrially relevant compounds, finding use and application in the food, pharmaceutical, cosmetic, and chemical industries. For example, it is expected that the salicylic acid (**2**) market size will grow exponentially by 2025, driven by increasing consumer inclination towards the health benefits of salicylic acid-concentrated cosmetic products. In 2018, the revenue generated from the cosmetic ingredient market globally was valued at over USD 500 billion [25]. The natural ferulic acid market size was over USD 35 million in 2018, and the industry expects consumption at over 750 tons by 2025 [26]. Ferulic acid is now widely used in the food and cosmetics industries. It is used as the raw material for the production of vanillin and preservatives, as a cross-linking agent for the preparation of food gels and edible films, and as an ingredient in skin protection agents [27]. Gallic acid has a diverse range of industrial uses, such as in the pharmaceutical, cosmetic, food and feed, ink and dye, and paper industries, among others [28].

Vanillic acid (**4**) is utilised as a pharmaceutical intermediate and as an important component of flavour and fragrances [29]. Nowadays, many compounds, including phenolic acids, can be produced using engineered microorganisms. These microbial cell factories can be cost-effective and environmentally friendly alternatives, delivering the yield and purity of compounds that are higher than those extracted from plants [30,31]. Notably, phenolic acids produced in plants are usually found in conjugated form, often covalently bound to carbohydrates [32], whereas the same phenolic acids produced by microorganisms are obtained as free compounds. The conjugated or free forms of these compounds are bioavailable and may be assimilated by humans [32,33].

This paper focuses firstly on natural sources and properties of phenolic acids. It introduces into methodologies used for the extraction of these bioactive compounds. The strategies for production of phenolic acids using conventional plant sources and metabolically engineered microorganisms are comparatively assessed. Metabolic engineering advances for biosynthesis of ferulic, gallic, salicylic (**2**), and *p*-coumaric (**26**) acids are exemplified. The development and use of new tools, such as clustered regularly interspaced short palindromic repeats associated protein 9 (CRISPR-Cas9) system and genetically encoded biosensors, to improve phenolic acid production is emphasised.

## 2. Chemical Structure and Overview of Phenolic Acids

Phenolic acids are typically divided into hydroxybenzoic and hydroxycinnamic acids. The phenolic acids and their structural characteristics are presented in Table 1 and Table 2. In addition, all hydroxycinnamic acids in plants and produced by microorganisms are usually found as *trans*- geometric isomers.

The structure of phenolic acids defined by the position and number of methoxy and hydroxy groups attached to the aromatic ring contribute to their antioxidant or anti-radical scavenging properties [34,35]. These bioactive compounds form phenoxyl radicals, whereas the hydroxyl groups help to stabilise them [36]. Subsequently, these phenoxyl radicals can positively contribute to the neutralisation of toxic free radicals in the human body. The more hydroxy and methoxy groups are bonded to the benzene ring, the better the determination of the radical scavenging properties [35], as well as the indirectly attached carboxyl group (e.g., –CH=CH–COOH) [37]. The radical scavenging activities decreases in the following order: gallic > gentisic > syringic > caffeic (**31**) > protocatechuic (**10**) > sinapic (**30**) > ferulic (**27**) > isoferulic (**29**) > vanillic > *p*-coumaric > *o*-coumaric (**24**) > *m*-coumaric (**25**) > salicylic > *p*-hydroxybenzoic (**1**) acid [35]. The content of phenolic acids and other phenolic compounds in the extracts is proportional to the free radical scavenging activity [34,38]. Therefore, the synergistic interactions between phenolic acids [39] or the other compounds influence the final antioxidant properties—the total phenolic content leads to higher antioxidant activities [39,40,41].

## 3. Phenolic Acids in Plants

### 3.1. Biosynthesis

**Figure 1:**
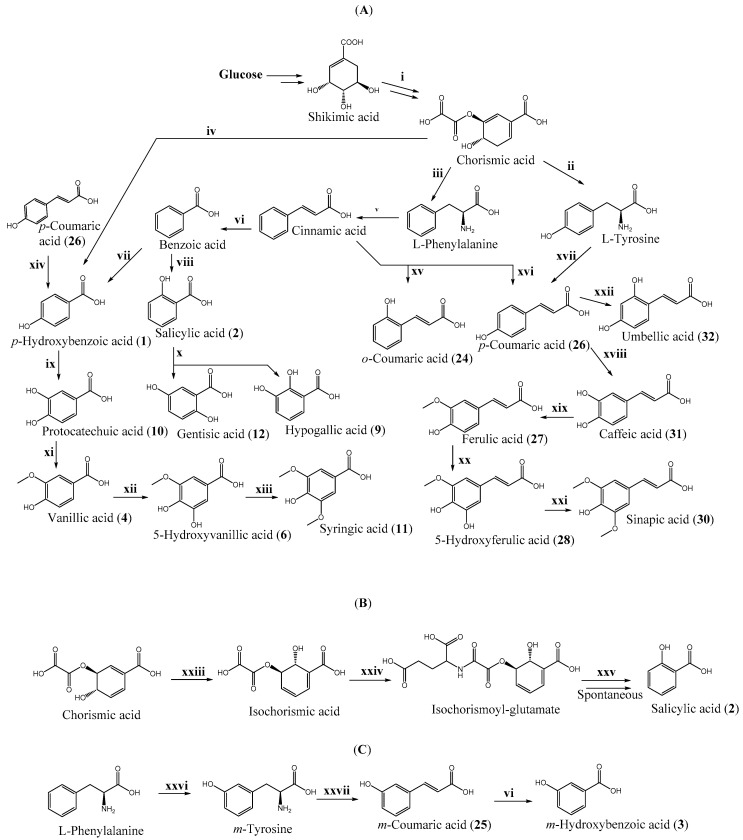
General scheme for the phenolic acid biosynthesis through the shikimate pathway (**A**), salicylic acid biosynthesis from isochorismate (**B**), and *m*-coumaric acid biosynthesis (**C**). Enzymes involved in the reactions: (i) shikimate kinase, 5-enolpyruvylshikimate-3-phosphate synthase and chorismate synthase; (ii) chorismate mutase, prephenate dehydrogenase; (iii) chorismate mutase, prephenate aminotransferase, arogenate dehydratase; (iv) chorismate-pyruvate lyase; (v) L-phenylalanine ammonia lyase (PAL); (vi) oxidase (or presumed β-oxidation); (vii) benzoic acid 4-hydroxylase; (viii) benzoic acid 2-hydroxylase; (ix) 4-hydroxybenzoic acid 3-hydroxylase; (x) salicylic acid 3-hydroxylase (S3H); (xi) protocatechuic acid 3-O-methyltransferase; (xii) vanillic acid 5-hydroxylase; (xiii) vanillic acid 5-O-methyltransferase; (xiv) 4-hydroxybenzaldehyde synthase and 4-hydroxybenzaldehyde dehydrogenase; (xv) cinnamic acid 2-hydroxylase; (xvi) cinnamic acid 4-hydroxylase; (xvii) tyrosine ammonia lyase (TAL); (xviii) *p*-coumaric acid 3-hydroxylase; (xix) caffeic acid 3-O-methyltransferase; (xx) ferulic acid 5-hydroxylase; (xxi) caffeic/5-hydroxyferulic acid O-methyltransferase (COMT); (xxii) *p*-coumaric acid 2-hydroxylase; (xxiii) isochorismate synthase (ICS); (xxiv) isochorismoyl-glutamate synthase (IGS); (xxv) isochorismoyl-glutamate A pyruvoyl-glutamate lyase (IPGL); (xxvi) cytochrome P450; (xxvii) tyrase.

Phenolic acids are generated from aromatic amino acids produced via the shikimate pathway (Figure 1) [42]. Here, the shikimic acid is converted into L-phenylalanine through a chorismic acid intermediate. The L-phenylalanine is converted into *p*-coumaric, salycilic, and *p*-hydroxybenzoic acids, which serve as precursors for other derivatives of phenolic acids. The hydroxylation and methylation of their aromatic ring result in biosynthesis of other hydroxycinnamic acids (e.g., ferulic and caffeic acids) or hydroxybenzoic acids (e.g., protocatechuic and *p*-hydroxybenzoic acids) [43].

In plants, enzymes involved in the shikimate pathway are localised in plastids (chloroplasts) [44]. Here, the phenylalanine, as well as chorismic, salicylic, and gallic acids, are synthesised [45,46], whereas other hydroxycinnamic and hydroxybenzoic acids are synthesised in the cytosol [47]. It is thought that hydroxybenzoic acids can be produced from structurally analogous hydroxycinnamic acids in coenzyme A (CoA) dependant (*β*-oxidative) or CoA-independent (non-*β*-oxidative) pathways or the combination of both them [48,49,50,51,52], which has been determined as occurring in peroxisomes [47] and mitochondria [53]. However, salicylic acid can also be synthesised in a few other ways: directly from either benzoic acid by benzoic acid 2-oxygenase or isochorismate by isochorismoyl-glutamate synthase and through the spontaneous or non-spontaneous (by isochorismoyl-glutamate A pyruvoyl-glutamate lyase) splitting of the isochorismoyl-glutamate A [54,55]. It has been observed that the concentration of salicylic acid in the cell usually depends on the isochorismate availability [56]. The conversion of salicylic acid into gentisic acid is catalysed by salicylic acid hydrogenase, with hypogallic (**9**) acid also produced in the reaction [57]. Isomers of *p*-coumaric acid are synthesised in plants in low amounts.

The biosynthesis of *o*-coumaric and *p*-coumaric acids co-occurs at microsomal membranes and is catalysed by cinnamic acid hydroxylases [58]. *m*-Coumaric acid could also be synthesised in small quantities from *m*-tyrosine that is produced from L-phenylalanine by radical hydroxylation under conditions of oxidative stress [59] or through direct hydroxylation, potentially involving the activity of cytochrome P450 enzyme, with the latter reported in *Festuca rubra* [60,61]. It is unknown if *m*-hydroxybenzoic (**3**) and *m*-coumaric acids can be synthesised from benzoic or cinnamic acids in plants. Therefore, the following hydroxylation of *p*-coumaric acids by *p*-coumaric acid 2-hydroxylase produces umbellic acid (**32**), which spontaneously forms lactone after closure of lactone ring under neutral or acidic conditions [62].

Orsellinic (**16**) and olivetolic (**23**) acids can also by synthesised in plants. The plant type III poliketide synthases such as tetraketide synthase from *Cannabis sativa* and orcinol synthase *Rhododendron dauricum* are involved in the biosynthesis of olivetolic and orsellinic acids as acetate-derived aromatic tetraketides, respectively [63].

In plants, phenolic acids are found in free, conjugated forms (as aglycones) and in bound forms that are attached to the cell walls [32,64]. Naturally occurring compounds such as hydroxy fatty acids, terpene alcohols, lignin, triterpenoids, or glucose can form ester or ether bonds with phenolic acids [65]. The soluble conjugates of phenolic acids are usually stored in the storage vacuoles, which may occupy up to 90% of the cell volume [66].

### 3.2. The regulation of Phenolic Acid Biosynthesis

The response to biotic stress can activate systemic acquired resistance (SAR) gene expression due to pathogen attack, or the interaction with beneficial microorganisms can activate the immunity system of the plant (induced systemic resistance (ISR)) [67]. The pathogen attacks cause biotic stress to plants, which results in the production of various reactive oxygen containing species (oxygen, hydroxyl radicals, hydrogen peroxide, superoxide molecules; ROS) and nitric oxide (NO), which are the most active in the alkaline pH, which results in the rise of cytosolic calcium ion concentration and closure of stomata [68,69].

The formed ROS radicals and NO result in the production of azelaic acid via the cleavage of double bond (C9) of unsaturated C18 fatty acids [70]. Azelaic acid induces the SAR genes, and the production of salicylic acid occurs when the isochorismate synthase 1 (*ICS1*) gene which is controlled by several transcription factors such as NAC transcription factor-like 9 (NTL9) and CCA1 hiking expedition (CHE), is activated [71]. Then, the synthesis of other metabolites is also activated, which leads to plant health improvements and acquired resistance. Produced salicylic acid can be deactivated by methylation and conversion into 2-methyl salicylic acid via the mediation of salicylic acid methyltransferase 1 (SAMT1), and the activation (the conversion to salicylic acid) of the latter compound is mediated by methyl salicylate esterase of salicylic acid-binding protein 2 (SABP2) [72,73].

In the interaction with beneficial microorganisms, the induced systemic resistance (ISR) system is regulated by several phytohormones such as salicylic acid, jasmonic acid, and ethylene [74]. Salicylic acid is an antagonist for jasmonic and ethylene, and their cross-talk is regulated by suppression of different transcription factors [74,75,76,77]. The plant response to this interaction leads to the strengthening of cell walls, closure of stomata, and other secondary metabolite production, or this response can be similar to pathogen attack [67].

The abiotic stress in plants results in the decline of energy supply because of inhibition of energy-releasing reactions and photosynthesis in chloroplasts [78]. The plant stress hormone abscisic acid is involved in all these regulations, and it is responsible for osmotic stress tolerance and plant water balance [79]. However, ROS, NO, and lipid molecules are also involved in the response of plants to abiotic stress, and activate induced systemic resistance (ISR) of plants.

### 3.3. Biotic and Abiotic Stress

The accumulation of both hydroxybenzoic and hydroxycinnamic acids has been observed as being elevated after treatment with fungal or bacterial pathogens. For example, gallic, syringic, *p*-coumaric, salicylic, and sinapic acids are accumulated in peas raised from seeds treated with *Trichoderma harzianum*, *Bacillus subtilis*, and *Pseudomonas aeruginosa* [80]. The produced amount of different phenolic acids varies depending on the pathogen.

There are several abiotic stress factors: salinity, metal ions, light, water scarcity, wounding, or elicitor treatment. The accumulation of hydroxybenzoic acids results from salinity, treatment with a metal ion, or UV light [81,82,83]. Wounding stress also activates the production of phenolic acid (especially ferulic acid) derivatives, and their concentration can be increased up to 100% [84]. The higher increase in the concentration of these compounds is obtained with a combination of wounding with hyperoxia, and chemical substances (elicitors) such as ethylene or methyl jasmonate [84,85]. Water deficit results in hydroxybenzoic acid biosynthesis [86]. The illumination by light with wavelengths less than 360 nm causes the isomerisation reaction of glucosides-bound *o*-hydroxycinnamic acid from *trans* to *cis* forms in leaves [87].

### 3.4. Factors Influencing the Amount of Accumulated Phenolic Acids

Several factors influence the accumulated and determined content of phenolic acids before or after the collection of raw materials. The seasonality, different cultivars, planting location, geographical area, agricultural conditions, part of the plant used (leaves, flowers, or whole plant), sample dryness (fresh or dried sample), and type of the plant may have an effect on the recovered amount of secondary metabolites including phenolic acids [88,89,90,91]. After collection of raw material, the drying temperature, storage temperature, and time, as well as the extraction method, the solvent used, and the number of extraction cycles, determines the final amount of obtained phenolic acids [92,93,94]. Notably, the storage temperature and time may lead to an increased concentration of total phenolic acids [93], whereas the increased oven-drying temperature leads to the decreased concentration of the same compounds [92].

## 4. Production and Extraction of Phenolic Acids from Plants and Algae

The concentrations of phenolic acids can vary significantly in various plants and algae. Crops, cereals, fruits and vegetables, oilseeds, herbs and spices, teas, and coffee are the major sources of phenolic acids. The highest amounts of these compounds obtainable from plants and algae are presented in Table 3. The quantity of free phenolic acids can range from micrograms to milligrams per gram of dry weight of plant tissue. The content of these compounds differs between sources. In the hydroxybenzoic acid group, the highest yields of amounts have been reported for gallic, gentisic, and syringic acids. The highest content of gallic acid varied between 1485–30603 µg/g dry weight (DW) in pulp from *Byrsonima ligustrifolia* fruits, followed by the diatom (*Odontella sinensis*; 9489 µg/g DW) and clove (*Eugenia caryophylata *Thunb.) leaves (7385 µg/g DW). The amount of gentisic acid has been reported to range from 120 to 8600 µg/g DW, with the highest amount extracted from lavender (*Lavandula officinalis* L.) flowers, followed by bitter melon (*Momordica charantia* L.) fruits (5910 µg/g). The highest concentration of syringic acid was found in blueberry (*Vaccinium. myrtillus* L.) fruits (5627.47 µg/g DW). In the hydroxycinnamic acid group, the highest yields were achieved for caffeic, ferulic, and *p*-coumaric acids. The amount of caffeic acid ranged from 537 to 17400 µg/g DW, with the highest amount extracted in roasted coffee beans, followed by tansy (*Tanacetum vulgare* L*.)* leaves (8940 µg/g DW), and potato (*Solanum tuberosum* L.) peels (3320 µg/g DW). Amounts between 116 and 7000 µg/g DW of ferulic acid were detected in white onion (*Allium cepa* L.) outer layers, baru (*Dipteryx alata Vog.*) nuts, hyssop (*Hyssopus officinalis* L.) herbs, mate (*Ilex paraguariensis* St. Hil.) leaves, and the bran and aleurone of wheat (*Triticum aestivum* L.). The content of *p*-coumaric acid varied between 2898-5265 µg/g DW in pulp from *Byrsonima ligustrifolia* fruits. Chokeberry (*Aronia melonocarpa* L.) fruits exhibited the highest amount of *p*-coumaric acid (4020 µg/g fresh weight (FW). The change in extraction method, solvents, and the application of hydrolysis may increase the determined phenolic acid concentrations in each plant.

### 4.1. Cereals

Crops contain various useful and bioactive compounds, including phenolic acids [92]. The most abundant phenolic acid in cereals is ferulic acid, consisting of up to 90% of total phenolic acid content in wheat, spelt, buckwheat, and oats [124]. It can be present at up to 2 mg/g in wheat bran and up to 7.98 mg/g in wheat aleurone [122,125]. Therefore, *p*-coumaric, ferulic, vanillic, and *p*-hydroxybenzoic acids are found in cereal grain, and the highest concentration of these compounds is located in husks or glume [124]. Changes in bound and soluble phenolic acids also occur during the development of wheat grain. The concentration of soluble hydroxycinnamic acids (ferulic acid, *p*-coumaric, and sinapic acids) steadily decrease, and the insoluble fraction increase during grain development, with these changes possibly influencing the properties of the cell wall and the whole grain [126].

### 4.2. Seeds and Oilseeds

Seeds such as chia, sunflower seeds, or flaxseeds are rich in bound phenolic acids (>10 mg/g) where the dominant acids are ferulic, *p*-coumaric, and caffeic acids, as well as protocatechuic and *p*-hydroxybenzoic acids and their derivatives (glucosides, chlorogenic acids, ferulic acid dehydrotrimers, monoacyl- and diacylquinic acids) [107,127,128]. However, monoacylquinic acids are the major compounds, consisting of bound caffeic and *p*-coumaric acids [127], which can be hydrolysed into their corresponding acids. Therefore, the free phenolic acids (gallic, sinapic, protocatechuic) were found in high concentrations only in canola seeds (52 mg/g defatted material, or up to 31 mg/g dry weight) [129,130]. Therefore, the seeds (sunflower seeds, chia) extracts contain the isomers of phenolic acids (*cis*- and *tran*s-ferulic acids, and *p*-, *o*-, *m*-coumaric acids) [107,127].

### 4.3. Fruits and Berries

One of the best sources of soluble phenolic acids is fruits and berries, which also contain free forms of these compounds [32]. Berries contain a concentration of soluble phenolic acids that is higher than fruits. The total amount of soluble phenolic acids is determined in plum, some varieties of apples, and cherry (up to 0.28 mg/g), but in rowanberry, chokeberry, blueberry, sweet rowanberry, and saskatoon berry, their concentrations reach up to 0.59−1.03 mg/g fresh weight [97]. Generally, berries and fruits contain high concentrations (up to 750 μg/g fresh weight for single phenolic acid) of gallic, ferulic, protocatechuic, caffeic, *p*-hydroxybenzoic, *p*-coumaric, sinapic, and chlorogenic (the source of bound caffeic acid) acids [32,97,131]. Therefore, trace amounts of 3- and 4-methyl salicylic acids (**19** and **20**) are found in blueberries [115].

In fruits, the concentration of phenolic acids depends on the part of fruit analysed. These compounds are usually located in the rind and peels, for example, the peel and rind of mangosteen fruit are rich in protocatechuic acid, but the peels of apples contain lower levels of phenolic acids than the whole fruit [97,99]. For berries, the change in concentration (increase or decrease) of phenolic acids depends on the maturation stages for a particular plant [96,132].

### 4.4. Vegetables

Vegetables contain more hydroxycinnamic acids than hydroxybenzoic acids. Sinapic acid is abundant in the *Brassicaceae* family (up to 79%–84% of all phenolic acids). In other vegetables such as red cabbage, carrots, lettuce, and artichoke, the predominant hydroxycinnamic acid is ferulic acid, with the total amount of phenolic acids reaching up to 0.52 mg/g fresh weight [81,133]. However, potatoes contain high amounts of gallic, *p*-coumaric, chlorogenic, and caffeic acids [134]. On the other hand, gallic and protocatechuic acids (hydroxybenzoic acids) are dominant in onions, and 3- and 4-methyl salicylic acids are found in beans [108,115].

The different layers of vegetables (onions, lettuce) accumulate diverse amounts of phenolic acids in which concentration depends on the plant type [135]. Peels or outer layers of roots contain the largest amount of phenolic acids. Notably, the treatment of vegetables by boiling, baking, or pickling may reduce the concentration of phenolic acids [133,136].

### 4.5. Nuts

Gallic, *p*-hydroxybenzoic, protocatechuic, and *p*-coumaric acids are the major bound phenolic acids found in nuts [112,123,137,138]. They are mainly localised in the protective skin [139]. The abundance of different phenolic acids depends on the part of the nut, for example, cashew kernels contain syringic and *p*-coumaric acids, but their testa is rich in gallic, syringic, and *p*-coumaric acids [112].

Roasting of nuts increases the content of gallic acid, which is released from hydrolysable tannins, but it may reduce the content of other phenolic acids [112,123]. The long term storage for different types of nuts also influences the phenolic acid content—*p*-hydroxybenzoic acid content increased up to 18-fold in almond skin while it did not influence the concentration of *p*-coumaric and *p*-hydroxybenzoic acids in peanuts [140,141].

### 4.6. Spices and Medicinal Herbs

Spices (basil, rosemary, star anise, chili pepper, bay, savory, etc.) used for culinary purposes are usually from the following families: *Lamiaceae*, *Lauraceae*, *Brassicaceae*, *Apiaceae*, *Labiateae*, *Schisandraceae*, *Myrtaceae*, *Solanaceae*, *Piperaceae*, and *Cinnamomum*. The same type of herbs can also be used in medicinal applications, but these must meet the specific quality standards [142]. The most popular medicinal herbs are from *Lamiaceae* and *Asteraceae* families, containing a high amount of phenolic acids and other phenolic compounds and showing better antioxidant properties when compared to other families, for example, the *Apiaceae* family [142]. Therefore, caffeic acid or the derivatives of this acid (rosmarinic or chlorogenic acids) are abundant in *Lamiaceae*, *Labiatae*, *Lauraceae*, and *Myrtaceae* families [103,110,143].

### 4.7. Tea, Cacao, and Coffee

The water extracts of cacao, tea leaves, and coffee are commonly used beverages in the human diet on an everyday basis. In coffee (roasted and unroasted), the major phenolic acids are derivatives of chlorogenic acid [37,144]. The roasting of coffee beans reduces phenolic acid content. The coffee brew from green beans contains up to 209 mg/100 g, and the roasting reduces the amount of phenolic acids by almost 2–4 times [37,144]. However, cocoa beverages contain a low amount of phenolic acids due to dilution, although cacao powder is rich in protocatechuic, vanillic, and syringic acids [97]. In teas such as black, mate, green, and oolong teas, the major phenolic acids are also chlorogenic acid derivatives [145]. Therefore, these teas (except mate) contain a huge amount of gallic acid (4.97–6.55 mg/g), but only mate tea contains caffeic, ferulic, and sinapic acids (0.76–1.87 mg/g) [104].

### 4.8. Algae

Gentisic, sinapic, caffeic, vanillic, gallic, ferulic, syringic, or *p*-hydroxybenzoic acids have been identified in the extracts of microalgae such as *Chaetoceros calcitrans*, *Isochyrysis galbana*, *Skeletonema costatum*, *Odontella sinensis*, *Phaeodactylum tricornutum*, and *Saccharina japonica* [102]. The highest total amount of these phenolic acids has been determined in *O. sinensis* (18.3 mg/g DW), where the dominant phenolic acid is gallic acid (9.5 mg/g DW).

### 4.9. Extraction of Phenolic Acids from Agro-Industrial Waste

Agro-industrial waste is composed of the organic residues that cannot be used for any purpose in their current state, and they are classified into several categories [146]. Agro-industrial residues are divided into industrial residues (e.g., peels of fruits and vegetables, oil cakes of soybean or other oilseeds, pomaces) and agricultural waste. Agricultural waste includes field-process residues remaining after crop harvesting (leaves, seedpods, stalks, or sterns), or they can be the residues of agricultural processing (straws, husks, bagasses, molasses, seeds, and roots). The amounts of each type of agro-industrial and agricultural waste (e.g., rice husk, apple pomace, citrus fruit processing residues) are generated in millions of metric tonnes per year worldwide [147]. These numbers show the huge importance of waste reprocessing to the value-added products for the utilisation of waste amounts.

Agro-industrial and agricultural waste contains mostly bound phenolic acids, which can be released after alkaline hydrolysis [148,149]. For the straws, techniques such as hydrothermal pretreatment are applied for enhanced release of bounded phenolic acids [150]. The amount of phenolic acids in an agro-industrial waste can reach up to a few percent of the raw material. For example, sugarcane bagasse contains up to 4.1% (or 41 mg/g) of *p*-coumaric acid [148]. The concentration of phenolic acids varies in the different types of waste. For mangosteen fruit, the amounts of total phenolic acids vary from 6 mg/kg DW in aril to 70 mg/kg (peel) and 218 mg/kg in rind [99]. For araticum fruit, the accumulation of free phenolic acids is higher in peels than seeds [109]. However, agro-industrial waste contains more variety of phenolic acids, including isoferulic, isovanillic (**5**), hypogallic, and γ-resorcylic (**15**) acids [151,152,153]. In the extracts, hydroxycinnamic acids (*p*-, *o*-coumaric, ferulic acids) can be found in both configuration as *cis* and *trans* stereoisomers, with the latter being the predominant form [148,150].

## 5. Production and Extraction of Phenolic Acids from Fungi

Although plants are the primary source, phenolic acids are also abundant as secondary metabolites in fungi such as mushrooms and yeasts [64]. As in plants, similar factors contribute to the biosynthesis of phenolic acids in fungi.

### 5.1. Mushrooms

Wild or cultivated mushrooms are popular dietary ingredients, and they can also be used in medicinal applications [154,155]. Hydroxybenzoic acids (*p*-hydroxybenzoic, protocatechuic, gallic, vanillic, and syringic acids), hydroxycinnamic acids (*p*-coumaric, *o*-coumaric, ferulic, caffeic acids), and phenolic acids derivatives (e.g., ellagic acid, chlorogenic acid, rosmarinic acid) are found in the extracts of mushrooms [38,156,157,158,159,160]. Most species of mushrooms accumulate a smaller amount of phenolic acids when compared with plants. The exceptions are *Ramaria botrytis*, which is rich in protocatechuic acid (343 μg/g DW) [156], and *Agaricus brasiliensis*, which contains high amounts (333–753 μg/g DW) of *p*-hydroxybenzoic, gallic, and ferulic acids [34]. The highest amounts of phenolic acids obtained from mushrooms are presented in Table 4.

β-Resorcylic (**14**) and protocatechuic acids were the phenolic acids identified in mushrooms with higher activity against the majority of Gram-negative and Gram-positive bacteria [161]. Methicillin-resistant *Staphylococcus aureus* (MRSA) was inhibited by β-resorcylic, vanillic, syringic (minimum inhibitory concentration (MIC) is 0.5 mg ml^−1^) and *p*-coumaric (MIC = 1 mg ml^−1^) acids. The presence of carboxylic acid (COOH), two hydroxyl (OH) groups in *para* and *ortho* positions of the benzene ring, and also a methoxy (OCH_3_) group in the *meta* position seems to be important for anti-MRSA activity [161].

### 5.2. Lichens

Lichens are symbiotic associations of algae and fungi or cyanobacteria living in amongst the filaments of various fungi such as *Trichoderma* spp., *Usnea*, and *Cladonia uncialis*. The lichenising fungi have polyketide synthase gene clusters, which enable them to produce orsellinic acid and its derivatives [163,164,165]. However, they can also produce unique phenolic acids. For example, compounds identified in *Usnea* extracts include phenolic acids such as everninic acid (**22**), *p*-orsellinic acid (**17**), *o*,*o*-dimethylorsellinic acid (**18**), and 3-butyryl-β-resorcylic acid [165]. Other alkylated orsellinic acid derivatives (*p*-orsellinic, ethyl-, propyl-, isobutyl, isopropyl-, and *sec*-butyl-orsellinates) were isolated from a *Parmotrema tinctorum* specimen [166], and these acids are specific to lichens.

Yeasts, as well as filamentous fungi, are usually used in fermentation processes, which are discussed below.

## 6. Production of Phenolic Acids Using Non-Modified and Engineered Microorganisms

### 6.1. Biosynthesis of Phenolic Acids from Organic Compounds Using Non-Modified Microorganisms

Natural microorganisms are capable of producing phenolic acids during assimilation and catabolism of other organic compounds (Table 5). For example, the biotransformation processes have been applied for the production of syringic acid from sinapic acid by *Paecilomyces variotii* [167]. Salicylic acid can be produced in high yields from naphthalene by *Pseudomonas aeruginosa* when the acidity of the medium is regulated by the addition of urea [168]. In addition, salicylic acid is produced in low yields from sucrose by *Rubrivivax gelatinosus* RASN4 strain isolated from rhizospheric soil of paddy fields [169]. The conversion of vanillic acid from ferulic acid (which is supplied as a pure compound or as an extract from pineapple peels) is performed by *Paenibacillus lactis*, *Halomonas elognata*, *Aspergillus niger*, and *Sporotrichum thermophile* [170,171,172,173]. The isolated *Aspergillus* spp. from moulds produces orsellinic acid also in less than 1% yield using Sabauraud medium [174]. Gallic acid is produced from tannic acid by *Kluyveromyces marxianus* and *Aspergillus fischeri* [175,176]. The product yields in these processes reach more than 90%.

### 6.2. Biosynthesis of Phenolic Acids from Organic Wastes Using Non-Modified Microorganisms

Microbial fermentation of waste is the most important processes because it allows for the reduction of the amount of organic waste and for the production of valuable bioactive compounds [177]. Microbial fermentation of waste can be carried out in a solid or liquid state, depending on the free floating water amount. Only the solid state fermentation can be performed by the limited quantity of microorganisms [183]. The substrate, desired products, and required enzymes determine the ability to employ the particular microorganism in each process. Other factors that influence the fermentation process are raw material; fermentation type (solid or liquid state); as well as temperature, dissolved oxygen concentration, pH, and microorganisms employed for the fermentation [177]. For this reason, the optimal fermentation conditions can lead to higher yields of phenolic acids, whereas inappropriate parameters (such as temperature) can even cause the death of the applied microorganisms [184]. In individual cases, other parameters can also be important. For example, sucrose concentration, MnSO_4_ concentration, and incubation temperature were the main parameters that significantly influenced phenolic acid production in palm oil mill effluent fermentation by *A. niger* IBS-103ZA [180]. The phenolic acids released during microbial fermentation can also have an inhibitory effect on the growth of microorganisms due to the increased quantity of hydrogen protons, which acidifies the cytoplasm of the microorganisms and inhibits metabolic functions [185].

Agricultural waste usually contains mostly lignin, cellulose, or hemicellulose, but also can contain gallotanins, tannic acids, various phenolic glucosides, and pectines. Depending on the raw material, researchers employ different microorganisms for the fermentation process. Some examples of these processes are presented in Table 5. *Bacillus* sp., *Rhodococcus jostii*, *Pandoreae* sp., and white-rot fungi can catabolise the lignin-containing waste and produce phenolic acids as intermediate compounds [186,187,188]. Therefore, some microorganisms such as *Acetoanaerobium* sp. WJDL-Y2 can produce ferulic and syringic acids from lignin as the final metabolites [189]. Ferulic acid from pectine of sugar beet pulp can be released by *Penicillium chrysogenum* [181]. It was determined that the produced ferulic acid esterase from *P. chrysogenum* can even hydrolyse hydroxybenzoic acid methyl esters [181]. Similarly, *Rhizopus oryzae* fungus was employed for the fermentation of rice bran, after which the greatest change in concentration was observed for ferulic acid (from 33 mg/g to 765 mg/g) [178]. Food-grade fungus *Lentinus edodes* CY-35 produces high levels of extracellular β-glucosidase, which is the major enzyme responsible for hydrolysing phenolic glucosides from cranberry pomace for the production of free gallic, *p*-hydroxybenzoic, and *p*-coumaric acids [190]. Only lactic acid bacteria such as *Lactobacillus arizononas* R13, *Lactobacillus plantarum* FST1.7, *Lactobacillus reuteri* R2, and *Weisella cibaria* PS2 can produce vanillic acid (up to 4.3 mg/L), *p*-hydroxybenzoic acid (up to 1.5 mg/L), *ρ*-coumaric acid (up to 14.0 mg/L), and salicylic acid (up to 9.9 mg/L) from the glucose using de Man, Rogosa and Sharpe (MRS) broth [191]. Lactic acid bacteria can also release *p*-coumaric acid, as well as caffeic and salycilic acids from grass [192]. However, natural fermentation (or composting) can also have a negative influence, causing the reduction in the concentration of phenolic acids (caffeic, vanillic, gallic), as reported in olive cakes [149].

### 6.3. Food and Beverage Enrichment with Phenolic Acids

Microbial fermentation is widely applied in the food industry. Alongside main fermentation products such as ethanol or lactic acid, it can generate various secondary metabolites, including phenolic acids. For example, after wine fermentation, the total amount of phenolic acids increases 1.5–2.0-fold because of increased concentrations of hydroxybenzoic acids (gallic, protocatechuic, vanillic, *p*-hydroxybenzoic, gentisic acids) [193]. Wheat bread doughs are enriched with phenolic acids, while the quality and properties of the doughs depend on the phenolic acid composition and amount [194]. *L. plantarum* strains LB126, 29DAN, and 98A are able to enrich the doughs in ferulic acid up to 400 µg/g DW, whereas the concentration of other phenolic acids such as caffeic, sinapic, *p*-hydroxybenzoic, and gallic acids is much lower (0.4–23.0 µg/g DW) [195]. *R. oryzae* RCK2012 has been used in wheat fermentation, enabling enrichment of final substance with *p*-hydroxybenzoic and vanillic acids [196].

### 6.4. Engineered Microorganisms for Phenolic Acid Production

Engineered microorganisms can be an alternative for the production of phenolic acids, which can be toxic compounds for microorganisms in low or high concentrations [197,198]. For this reason, the processes for the production of phenolic acids in high yields can be developed by the overexpression of efflux proteins or the use of microorganisms that are naturally resistant or show a high tolerance to the phenolic compounds and to the whole biochemical reactions that take place in organisms [198,199,200]. Some bacteria (*Esherichia coli*, *Pseudomonas putida*, *Corynebacterium glutamicum*) and yeast (*Saccharomyces cerevisiae)* can be genetically modified for the production of different phenolic acids. Both bacteria and yeasts are relatively economically feasible, fast-growing systems that can be cultured in bioreactors with high cell density [200].

#### 6.4.1. Shikimate Pathway in Engineered Microorganisms

**Figure 2:**
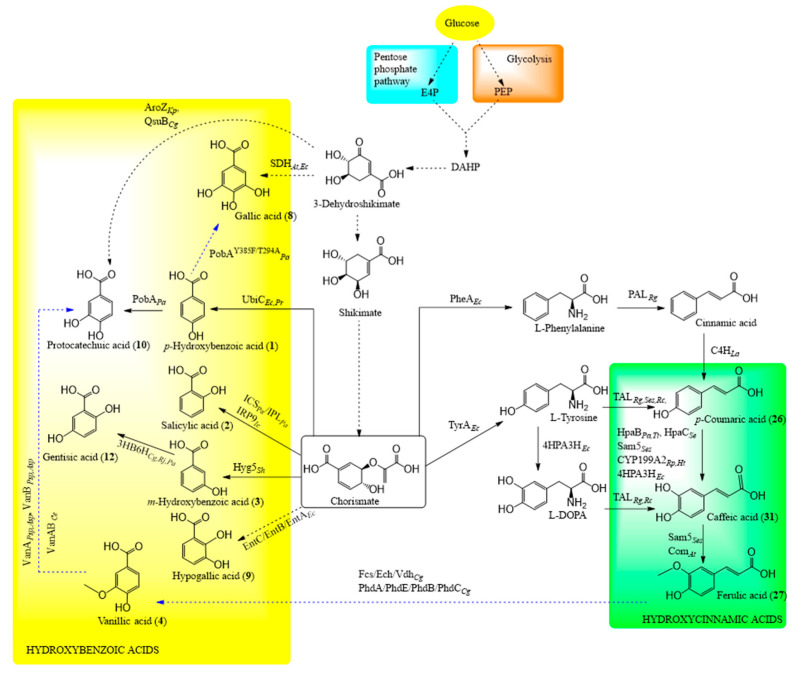
General pathways for the biosynthesis of phenolic acids by the engineered microorganisms. Solid arrows indicate the direct conversion, and the dotted arrows indicate the conversion through two or more reactions. Black arrows show phenolic acid production from glucose while blue arrows indicate their production from precursors. Abbreviations: aroZ, dehydroshikimate dehydratase; C3H, 4-coumarate 3-hydroxylase; C4H, trans-cinnamic acid 4-hydroxylase; COMT5, caffeic acid 3-*O*-methyltransferase; CYP199A2, cytochrome p450 CYP199A2; 3,5-DHS, 3,5-dehydroshikimate; 3-DHS, 3-dehydroshikimate; E4P, erythrose-4-phosphate; Ech, enoyl-coenzyme A hydratase/aldolase; EntA, 2,3-dihydro-2,3-dihydroxybenzoic acid dehydrogenase; EntB, isochorismatase; EntC, isochorismate synthase; Fcs, feruloyl-CoA synthase; HpaB and HpaC, 4-hydroxyphenylacetate 3-monooxygenase oxygenase components; 4HPA3H, 4-hydroxyphenylacetate 3-hydroxylase; 3-HBA, m-hydroxybenzoic acid; 4-HBA, 4-hydroxybenzoic acid; 3HB6H, 3-hydroxybenzoate 6-hydroxylase; Hyg5 encodes chorismatase/3-hydroxybenzoate synthase; L-DOPA, L-3,4-dihydroxyphenylalanine; ICS, isochorismate synthase; IPL, isochorismate pyruvate lyase; IRP9, salicylate synthase; PAL, phenylalanine ammonia-lyase; PEP, phosphoenolpyruvate; pheA, chorismate mutase/prephenate dehydratase; PobA, p-hydroxybenzoate hydroxylase; PhdA, acyl-CoA ligase; PhdB, 3-hydroxyacyl-CoA dehydrogenase; PhdC, 3-oxoacyl-CoA ketohydrolase; PhdE, enoyl-CoA hydratase; QsuB, 3-dehydroshikimate dehydratase; Sam5, 4-coumarate 3-hydroxylase; SDH, shikimate dehydrogenase; TAL, tyrosine ammonia lyase; tyrA, prephenate; UbiC, chorismate lyaze; VanA and VanB ( or VanAB), the terminal oxygenase (VanA) and the reductase (VanB) are subunits of the vanillate-O-demethylase; Vdh, vanillin dehydrogenase. The enzymes/genes sources: *Asp*, *Acinetobacter spp; At*, *Arabidopsis thaliana; Ce*, *Corynebacterium efficiens; Cg*, *Corynebacterium glutamicum*, *Ec*, *Esherichia coli; Ht*, *Helianthus tuberosus;* *Kp*, *Klebsiella pneumoniae; La*, *Lycoris aurea;* *Pa*, *Pseudomonas aeruginosa;* *Pr*, *Providencia rustigianii; Psp*, *Pseudomonas spp; Rc*, *Rhodobacter capsulatus;* *Rg*, *Rhodotorula glutinis; Rj*, *Rhodococcus jostii; Rp*, *Rhodopseudomonas palustris; Se*, *Salmonella enterica; Ses*, *Saccharothrix espanaensis;* *Sh*, *Streptomyces hygroscopicus; Tt*, *Thermus thermophilus;* *Ye*, *Yersinia enterocolitica.*

The microbial biosynthesis of phenolic acids is rather similar to the reactions occurring in the plants. Engineered microorganisms are mostly developed to produce phenolic acids via the shikimate pathway (Figure 2), which is the primary biosynthetic route for synthesising aromatic amino acids and their derivatives [201,202]. Microorganisms can be engineered to utilise different carbon sources, renewable sugars, glucose, xylose, or glycerol or precursors for production of these compounds [198,203]. The precursors can be other phenolic acids or amino acids [204,205]. Therefore, the product yields in microbial synthesis are ultimately limited by the mechanism utilised for glucose transport [206]. The condensation of glycolytic intermediate phosphoenolpyruvate (PEP) and pentose phosphate pathway intermediate erythrose-4-phosphate (E4P) allows for the generation of shikimate, which is converted to chorismate. Chorismate is the major intermediate in this shikimate pathway. All transformations of chorismate can be performed with different native or engineered enzymes obtained from bacteria, yeast, fungi, or plants.

#### 6.4.2. Biosynthesis of Hydroxycinnamic Acids

*p*-Coumaric acid can be obtained from cinnamic acid [207] or tyrosine [204] using glucose as a carbon source [208]. Cinnamic acid is usually produced from phenylalanine. L**-Phenylalanine or L**-tyrosine are formed from chorismate or directly added into the media as precursors. The extension of this pathway leads to the production of caffeic acid and ferulic acid [209]. The conversion of coumaric acid to caffeic acid can be mediated by 4-hydroxyphenylacetate 3-hydroxylase (*4hp3h*), 4-coumarate 3-hydroxylase encoded in *sam5*, 4-hydroxyphenylacetate 3-monooxygenase (*hpaC* and *hpaB*), or cytochrome P450 [210,211,212,213,214]. Therefore, for the latter, it is difficult to express in the bacterial systems and requires the co-expression of redox partners—the putida redoxin reductase gene (*pdR*) and the palustris redoxin gene (*pux*) from *Rhodopseudomonas palustris* in the host organism [210,215]. Caffeic acid is also produced from tyrosine through 3,4-dihidroxyphenylalanine (L-DOPA) with mediation by the enzymes 4-hydroxyphenylacetate 3-hydroxylase (4HPA3H) and tyrosine ammonia lyase (TAL) [211]. Caffeic acid 3-*O*-methyltransferase mediates the methylation of caffeic acid to ferulic acid [216]. Sinapic acid is produced through the oxidation of sinapaldehyde by *E. coli* after expressing gene *ref1*, which encodes a sinapaldehyde dehydrogenase required for sinapic acid and sinapate ester biosynthesis [217]. The latter modified bacterium was also able to convert coniferaldehyde to ferulic acid as well.

#### 6.4.3. Biosynthesis of Hydroxybenzoic Acids

Chorismate is converted directly to the *o*-, *m*-, and *p*-hydroxybenzoic acids [218]. Gentisic and protocatechuic acids are obtained from the *m*- and *p*-hydroxybenzoic acids, respectively, after hydroxylation. Gallic acid is synthesised from 3-dehydroshikimate with the mediation of shikimate dehydrogenase or from *p*-hydroxybenzoic acid with mediation by mutant hydroxylase [219,220]. Hypogallic acid can be produced in *E. coli* when chorismate is first converted to isochorismate, then to 2,3-dihydroxy-2,3-dihydrobenzoic acid (2,3-dihydro-2,3-DHBA), and finally to this phenolic acid by the mediation of isochorismate synthase, isochorismatase, and 2,3-dihydro-2,3-DHBA dehydrogenase, respectively [221]. Protocatechuic acid is synthesised from ferulic acid through non-β-oxidative or β-oxidative CoA-dependent pathways, where the last step is the biotransformation of vanillic acid mediated by vanillate O-demethylase [222,223]. Vanillic acid is synthesised from ferulic after disruption of *vanA* and *vanB* genes from *Pseudomonas fluorescens* and *P. putida* using *p*-coumaric acid or ferulic acid extracted from corn bran [205,224]. Syringic acid is produced during oxidation of syringaldehyde by the engineered *E. coli*, after insertion of *desV* and *ligV* genes [225].

The secondary metabolites of fungi (6-methyl salicylic (**21**), orsellinic and α-resorcylic (**13**) acids) and olivetolic acid can be produced as the final compounds from coenzymes (malonyl-CoA, acethyl-CoA, propionyl-CoA, hexanoyl-CoA) of acetyl-malonate or propionyl pathways, after expression of polyketide synthase genes in engineered microorganisms. α-Resorcylic acid is biosynthesised from maltose in *Aspergillus oryzae* RIB40 after overexpression of tetraketide alkyl-resorcinol/resorcylic acid synthase *csyA* gene under the control of the promoter *amyB* in *A. oryzae* [226]. The expression of phosphopantetheinyl transferase gene *npgA* of *Aspergillus nidulans* and 6-methylsalicylic acid synthase (6-MSAS) gene *atX* from *Aspergillus terrus* in *Pichia pastoris* resulted in the production of 6-methylsalicylic acid from methanol [227]. Later, the overexpression of the *hrk1* gene, which is responsible for the tolerance and acetyl-CoA synthetase ScAcs1 from engineered *P. pastoris* in *K. phaffii,* resulted in the production of 6-methyl salicylic acid from acetate [228]. 6-Methylsalicylic acid synthase, which consists of a few domains (such as ketoacylsynthase, acyltransferase, thioester hydrolase, ketoreductase, and acyl carrier protein), is activated by phosphopantetheinylation, and then it is able to catalyse the synthesis of 6-methyl salicylic acid from one acetyl-CoA and three malonyl-CoA under consumption of one dihydronicotinamide-adenine dinucleotide phosphate (NADPH) via oxidation and decarboxylation reaction [229,230]. High titer of 6-methyl salicylic acid (440.3 mg/L) was achieved by the production of engineered *E. coli* from glycerol in fed-batch fermentation after overexpression of 6-methyl salicylic acid synthase gene from *Penicillium griseofulvum* and the *accBCD1* gene from *C. glutamicum* and knocking down the pabA gene [231]. Orsellinic acid was also produced after the expression of the BY1 gene from basidiomycetes in *A. niger* with the mediation of non-reducing polyketide synthases *pks1* and *pks2* [232]. For the production of olivetolic acid in engineered *E. coli*, the olivetolic acid synthase (*ols*) and cyclase (*olc*) genes from *C. sativa* were inserted for the synthesis of this compound from hexanoyl-CoA and malonyl-CoA [233]. For the biosynthesis of hexanoyl-CoA, the module of the β-oxidation reversal was also engineered. After optimisation of temperature, inducer concentrations of isopropyl β-d-1-thiogalactopyranoside (IPTG) or cumate, and working volume, the engineered strain produced 80 mg/L from glycerol suplemented with hexanoate in the first 24 h [233].

Some examples from literature [199,204,205,206,207,208,209,210,214,217,218,221,224,225,226,227,228,231,233,234,235,236,237,238,239,240,241,242,243,244] of the highest production of phenolic acids biosynthesised by the engineered microorganisms are presented in Supplementary Materials Table S1. However, there are no data on the production of other phenolic acids, such as isovanillic, *o*-vanillic (**7**), and isoferulic acids and isomers of *p*-coumaric, 6-methyl salicylic, and α-resorcyllic acids, by the engineered microorganisms.

## 7. Techniques for Extraction and Analysis of Phenolic Acids

The extraction of phenolic acids for large scale production is a complex process. An extraction scheme typically involves four stages: pre-treatment (or preparation) of material, extraction, purification, and analysis of the obtained extracts.

### 7.1. Preparation of Material

Before the extraction of phenolic compounds from either fresh or dried raw biomass, a pre-treatment step is typically performed. The biomass is usually milled or ground to reduce the size and increase effective surface area. For efficient recovery of phenolic acids, we can remove non-polar compounds such as lipids, chlorophyll, and steroids can from samples before the extraction. Defatting is achieved with the use of non-polar solvents such as hexane, supercritical carbon dioxide, and others.

### 7.2. Extraction

The defatted plant feedstocks are then ready for the extraction of phenolic acids with different polar or partially polar solvents such as methanol, ethanol, water, acetone, or ethyl acetate. For extraction of bound or conjugated phenolic acids—enzymatic or chemical—by an acid or base, we can perform hydrolysis. Conjugated forms of phenolic acids, for example, are more likely to be released by alkaline hydrolysis rather than hydrolysis in acidic conditions [245]. Frequently, ester or ether forms of bound phenolic acids are extracted in higher amounts with alkaline (pH ≥ 7) or acidified (2 ≤ pH < 7) solvents, respectively [246]. For the prevention of caffeic acid degradation under alkaline conditions, we can use citric acid and ethylenediaminetetraacetic acid (EDTA) [247]. Enzyme-assisted extraction with various natural or recombinant enzymes, such as esterases, pectinases, tannases, or xylanases, can also enable the release of phenolic acids from plants [248,249,250,251,252,253,254,255]. This method alone is used to produce phenolic acids from waste instead of the use of solid (or liquid) state fermentation with microorganisms.

Several different extraction techniques have been suggested for recovery of phenolic acids from various matrices. Depending on whether the solvent is required, we can divide these techniques into solvent-less extraction techniques and solvent-based techniques. Solvent-less extractions use conventional presses and modern techniques such as microwave hydrodiffusion and gravity (MHG), pulsed electric fields (PEF), instant controlled pressure drop (DIC), and others [256].

Solvent-based techniques can be further categorized into solid–liquid extraction (SLE) and liquid–liquid extraction (LLE). There are a few commonly used SLE techniques. Maceration and percolation are the simplest forms of SLE that can be performed with cold or hot solvents. Pressurised liquid extraction (PLE) combines high temperature and pressure with liquid solvents to quickly and efficiently extract the analytes from the solid matrix [257]. In ultrasound-assisted extraction (UAE), cavitation effects, which occur during ultrasonic irradiation, lead to enhanced extraction of targeted analytes via a series of mechanisms including fragmentation, erosion, capillarity, detexturation, and sonoporation [258]. The supercritical fluid extraction (SFE) is performed with liquid carbon dioxide with or without the addition of co-solvents at high pressure. Other non-conventional extraction techniques that have been suggested include microwave-assisted extraction (MAE), high-voltage electrical discharges, and high-hydrostatic pressure. Two or more extraction techniques can be combined for the extraction of phenolic acids since each method has its advantages and disadvantages. For example, maceration and percolation do not require high investment or operation costs but require more time [259] and solvents when compared with PLE. Moreover, increased yields of recovered phenolic acids have been typically reported with non-conventional techniques such as MAE, UAE, and PLE. Nevertheless, for any extraction method, the recovery of phenolic acids significantly depends on the type of solvent, temperature, time of extraction, solid to solvent ratio, and the stability of phenolic acids under the applied extraction conditions [259,260,261,262]. For example, in MAE, the phenolic acids containing fewer substituents in the aromatic ring or methoxy instead of hydroxyl group exhibit higher stability than hydroxyl group-rich compounds [263]. On the other hand, in UAE, protocatechuic, *p*-hydroxybenzoic, vanillic, *p*-coumaric, and ferulic acids are stable up to 65 °C, while sinapic and caffeic acids degrade under ultrasound treatment [264].

### 7.3. Separation

Typically, the obtained crude extracts will contain a mixture of different phenolic acids as well as other compounds (e.g., flavonoids, pigments) [101,116,265]. If necessary, the crude extracts can be further fractionated or purified by means of preparative chromatographic techniques (i.e., preparative high pressure liquid chromatography (HPLC), thin-layer, flash, or counter-current chromatography (CCC)) or other techniques such as liquid–liquid extraction (LLE) and solid phase (SPE) extraction [266]. In these cases, compounds of interest can be separated according to their polarity, affinity to the stationary phase, or their solubility in one of the two immiscible solvents. Typical solvents in LLE include water and a nonpolar organic solvent (e.g., diethyl ether) or the use of ionic liquids [267,268]. In SPE, depending on the physicochemical properties of analytes, elution of the desired analytes of interest or undesired impurities in the sample from the stationary phase can be achieved with various solvents. In matrix solid-phase dispersion, a variation of SPE samples can be mixed with solid sorbents and solvents for simultaneous preparation, extraction, and fractionation of solid, semi-solid, or highly viscous biological samples. For this method, the selection of the most appropriate elution agent and the volume of elution media are necessary to be determined in order to achieve a high recovery of phenolic acids [269].

### 7.4. Analysis of Phenolic Acids

Various chromatographic techniques can perform qualitative and quantitative analysis of the extracted compounds including phenolic acids. For phenolic acid analysis, the method of choice are HPLC systems, which can be coupled to detectors such as ultraviolet light, diode array, fluorescence, or mass spectrometer (MS). Samples can also be analysed, typically after derivatisation, by gas chromatography (GC) systems coupled to flame ionisation, electron capture, or mass spectrometry detection. Other suggested techniques for the analysis of phenolic acids include CCC or capillary electrophoresis (CE) and nuclear magnetic resonance spectroscopy (NMR). CCC is advantageous due to its low cost, low solvent consumption, low risk of sample denaturation, minimised tailing of the chromatograms, and lack of irreversible absorption or loss of the substances injected into the system [270]. On the other hand, CE can be chosen due to the minimal sample volume required, short analysis time, and high separation efficiency [271]. NMR, on the other hand, offers a unique advantage over the above-mentioned techniques for structural characterisation of purified complex or novel natural products. Besides the analysis of isolated compounds, NMR-based metabolomics have gained popularity over the last two decades with, however, less sensitivity as compared to LC- or GC-based metabolomics [272].

### 7.5. Alternative Methods for Quantification of Phenolic Acids: Biosensors

The biosensor approach is an alternative to the analytical methods described above for the quantification of phenolic acids. Typically, bio-sensors consist of three components: recognition element (bioselective membrane); physical transducer; and electronic system for signal amplification, recording, and data representation [273]. Biosensors can be classified according to the type of transducer, or the nature of the biological entity of the recognition element [273]. Transducer-type biosensors can be electrochemical (ampiometric and potentiometric), electrical (conductometric and ion-sensitive), optical, piezoelectric (mass detection methods; acustic and microcantilever), and thermal detection [273]. The biological entity can be an enzyme, nucleic acid, antibody, hormone, lectine, cell structure, or tissue, but for phenolic acids, bio-sensors enzymes, and cells are applied. These components interact specifically with target compounds, and as a result, the occurred biochemical reaction is transformed into measurable signal through transducer.

Biosensors can find important applications for phenolic acid determination in the food and nutraceutical industry, or for environmental screening. The major types of biosensors of phenolic acid monitoring are discussed below.

#### 7.5.1. Enzyme-Based Biosensors

Enzyme-based biosensors are typically constructed using highly active extracellular enzymes from fungi or bacteria, which are immobilised on the surface of the electrode (e.g., graphite electrode). Immobilisation of enzymes on the electrode surface is one of the critical steps that determine the effectiveness of the enzymatic biosensor by preserving the specificity and native structure of the enzyme [274]. Different enzymes such as laccases, tyrosinases, laccase-tyrosinases, or peroxidases are applied for the determination of phenolic acids or polyphenols in beer, wine, honey, or extracts [275,276,277,278]. Enzyme-based biosensors are amperometric forms and can be sensitive not only to simple molecules, such as caffeic acid, but to more complex structures. For example, the use of laccase from white-rot fungi *Cerrena unicolor* allowed the construction of a biosensor for caffeic acid monitoring in a flow-injection system, which can also be applied to detect more complex compounds, with three or more aromatic rings with different sensitivity [276]. Amperometric biosensors are known to have only a narrow linear range for the specific compounds in the μmol/L region.

#### 7.5.2. Transcription Factor-Based Biosensors

Transcription factor (TF)-based gene expression systems can be applied as genetically encoded biosensors for the detection and monitoring of various metabolites [279,280], including phenolic acids. TF-based biosensors are composed of a transcription factor-based inducible gene expression system, which regulate the correct level of gene expression in the engineered microorganism that responds to the specific compounds such as one or more phenolic acids. TF-based biosensor structure consists of sensor–promoter–reporter genes. The use of a fluorescent reporter gene enables the monitoring of the concentration of the analyte by fluorescence output, proportional to the concentration, easily and rapidly. Sensor–reporter systems are used for real-time monitoring and high-throughput screening of phenolic acids, whereas sensor-actuators are used for adaptive laboratory evolution and dynamic pathway control [281].

For many phenolic acids, TF-based biosensors have been developed, and they are listed in Table 4. The major application of these biosensors is for sensing of compounds produced in lignin valorisation. The transcription factors of LysR, MarR, PadR, AraC/XylS, and IclR families are applied in repressor- and activator-type biosensors using different host organisms such as *E. coli*, *P. putida*, *Pseudomonas fluosrescens*, *S. cerevisiae*, and *C. glutamicum*. The dynamic ranges of other biosensors reach from 1- to over 200-fold (Table 6) when the concentration of analyte does not exceed 20 mM. TF-based biosensors specific to salicylic acid and *p*-coumaric acid have been shown to possess the highest dynamic range of more than 100-fold [282,283,284]. The repressor-type TF-based biosensors have more flexibility in changing the TF binding site position in the reporter promoter, which is impossible for the activator-type TF-based biosensors [279]. Therefore, activation-type biosensors are easier and more convenient to use because the signal is directly proportional to the concentration of the activator. The specificity of constructed biosensors depends on the purpose of the biosensor, and some of them are designed to sense structurally similar compounds [285,286].

## 8. Future Perspectives and Limitations of Phenolic acid Production

The inhibition effect of different compounds and the rate-limiting steps reduce the yield of targeted compounds. For example, for caffeic acid production from kraft pulp hydrolysate by *E. coli* YD01, furfural or syringic acid acted as inhibitors and reduced caffeic acid production by 20% without, however, reducing the cell growth at high concentrations [297]. The engineered *E. coli* strain, which converts *p*-coumaric acid to caffeic acid in the presence of flavin adenine dinucleotide (FAD) and nicotinamide adenine dinucleotide hydride (NADH), accumulated L-dopa and *p*-coumaric acid [211]. This accumulation was considered as a rate-limiting step.

### 8.1. Improved Phenolic Acid Production through Engineering Microorganisms

Some phenolic acids such as vanilic, ferulic, caffeic, and gentisic acids are obtained in rather low concentrations by engineered microorganisms. On the other hand, others such as protocatechuic, hydroxybenzoic, *p*-coumaric, and salicylic acids can be produced in high concentrations. Nevertheless, obtained product titers are still not appropriate for the economically feasible production and application in large scale reactors. Usually, the number of differentially expressed genes and proteins involved in aromatic amino acid biosynthesis, their catabolism, and transport system are linked to the improved production of phenolic acids [298]. There are a few possible strategies for enhancing the production of desired phenolic acids in the engineered microbial cell factories, including better carbon flux control (or reduction of by-products formation), improving the product transport, increasing of the specific growth rate of the cell, or using the platforms for the utilisation of waste. The optimisation of various parameters and the use of different carbon sources could also be important for the production of phenolic acids. The optimum enzyme expression levels can be controlled by the construction of ribosome-binding sites and transposon insertion libraries [284].

### 8.2. Transporters

The intracellular and extracellular concentrations of the products or intermediates influence the obtained product concentration. The transport systems influence the intracellular concentration of phenolic acids in the engineered microorganism, and it can be related to lowering the toxicity of the products inside the cell or optimising the carbon flux. The membrane protein overexpression can lead to the creation of efflux systems where the transcription levels can result in mutations in the promoter or a slower uptake of IPTG, and it is regulated by inhibitors [299]. The different promoters influence phenolic acid production—P_T7,_ for example, performed better than P_tac_ and led to higher salicylic acid titer (233.6 ± 6.6 mg/L) by *E. coli* [237]. The expression of transporters is affected by the carbon source [298]. Therefore, the use of heat shock promoters of *dnaK* and *ibpA* enabled higher *p*-coumaric acid (119.6 μM) and caffeic (13.7 μM) acid production in *E. coli* [204].

The deletion of unfavourable transporters results in the increased titers of the products. For example, the deletion of tryptophane amino-acid transporter 1 (TAT1), resulted in the increase of *p*-coumaric acid titer in 50% in biosynthesis by *S. cerevisiae* [197]. The knockouts of polyamine and arginine transporters (*tpo1* and *alp*), as well as deletion of transporters of amino acids (bap2, agp3), acetate (ady2), and galactose (gal2), gave a lower improvement in *p*-coumaric acid biosynthesis [197].

### 8.3. Product Removal

The extracellular concentration of the products can be lowered by employing resin-based extraction. For example, the engineered *E. coli* could synthesise 41 g/L of protocatechuate under glucose-rich conditions in 48 h [239]. The culture medium pumping through the column filled with resin allowed for the control of the maximum concentration of protocatechuate in the media. The final concentration of this compound (eluted from resin and extracted from culture media) was 71 g/L [239].

### 8.4. Availability of Tools

Transposon mutagenesis and codon optimisation techniques were applied for vanillic, caffeic, and *p*-hydroxybenzoic acid production in engineered microorganisms [205,214,233,236]. For successful *p*-coumaric acid biosynthesis, researchers applied high-throughput sequencing technology and gene overexpression via the CRISPR*/Cas9* system [207,208]. The overexpression of endogenous yeast genes using the CRISPR*/Cas9* system and optimisation of carbon distribution between glycolysis by replacing the several promoters of its genes in *S*. *cerevisiae* resulted in the highest production of *p*-coumaric acid (12.5 g/L) from glucose under fed-batch conditions [208].

Biosensor-guided improvements were applied for salicylic acid production [284]. In this example, the genes (*entC*, *pchB*, *aroL*, *ppsA*, *tktA*, *aroG^fbr^*) required for salicylate production were overexpressed, and Ara-SA sensor system was constructed in *E. coli*. AraC-based sensor system controlled the expression of β-galactosidase (*lacZ*), cleaving X-gal and generating a blue colour. After optimisation of gene expression patterns, salicylic acid production was enhanced up to 123%. The transposon mutagenesis, screening, and deletion of gene encoding RNaseD (*rnd*) from host bacteria resulted in the additional increase of salicylate production by 27%. The highest salicylate concentration (16.5 mM) was obtained after 48 h induction.

### 8.5. By-Products

The carbon flux control toward the shikimate synthesis pathway can reduce the formation of undesired side-/by-products, and it results in better yields of phenolic acids [234]. The deletion of glucose metabolism repressor *hexR* may increase the availability of shikimate pathway precursors E4P and the necessary reducing equivalents NADPH [300]. The deletion of genes *pobA*, *pheA*, *trpE*, *hexR*, and *tyrA* for carbon flux regulation stopped the accumulation of phenylalanine and tyrosine in *E. coli* or *P. putida*, and enhanced the concentration of *p*-hydroxybenzoic acid [237,300,301]. The deletion of *hexR* controlling glucokinase (*glu*), glyceraldehyde-3-phosphate (*gap-1*), and several Entner–Doudoroff pathway enzymes (*zwf*, *pgl*, *edd*, and *eda*) from *P. putida* KT2440 increased the flux toward pentose phosphate pathway and NADPH pool and resulted in the increased *p*-hydroxybenzoic acid production [300]. The reduction of by-product formation in *p*-coumaric acid-overproducing *S. cerevisiae* strain was carried out by knocking out phenylpyruvate decarboxylase ARO10 and pyruvate decarboxylase PDC5 and overexpressing of feedback-resistant 3-deoxy-D-arabinoheptulosonate 7-phosphate (DAHP) synthase and chorismate mutase together with overexpressing shikimate kinase enzymes from *E. coli*, homologous to the pentafunctional enzyme *aro1p* and to the bifunctional chorismate synthase-flavin reductase *aro2p* [243].

### 8.6. Enzyme Choice and Engineering

The different types and different sources of enzymes influence the yield of the final product due to their activity. The sources of natural enzymes and their genes were compared in gentisic and caffeic acid production. The gentisate production was influenced by *C. glutamicum* or *R. jostii* genes encoding for 3-hydroxybenzoate 6-hydroxylase [237]. The endogenous 4HPA3H in *E. coli* was more efficient than cytochrome P450 CYP199A2 for the conversion of *p*-coumaric acid to caffeic acid [244]. However, the mutant enzymes show higher activity than natural enzymes and allow high yields of phenolic acids to be obtained. For example, the expression of engineered genes of DAHP synthase and chorismate mutase in *E.coli* led to the production of *p*-coumaric acid in the highest titer (1.93 ± 0.26 g/L) [243]. Similarly, gallic acid was produced by *E. coli* during conversion of *p*-hydroxybenzoic acid by *p*-hydroxybenzoate hydroxylase with Y385F and T294A mutations [240]. Thus, the overexpressed gene copy number also matters. In *E. coli*, up to 10 copy numbers of TAL or medium copy number vector carrying *entCBA* gene performed best in the production of *p*-coumaric and hypogallic acids, respectively [209,221].

### 8.7. Fermentation Conditions and Substrates

Phenolic acid production is also affected by the cultivation condition (batch or chemostat cultivation) [197]; a sole or mixed carbon source [301]; and if the co-feeding of xylose, glucose, or glycerol is applied [203]. The lower purity of carbon source (crude or purified glycerol used) has a positive effect on product yields [302]. The optimisation of culture medium with yeast extract (from 1 g/L to 5 g/L) resulted in the increased production of *p*-coumaric acid by engineered *E. coli* [242].

### 8.8. Application of Other Methods

The application of co-culture technique could enable the production of many phenolic acids from glucose. For example, the L-tyrosine-overproducing strains [303] could be applied for precursor synthesis, which could be assimilated by the modified microorganism for the production of *p*-coumaric, ferulic, or caffeic acids. The growth rate of bacteria can be improved by the introduction of the transcriptional regulators *gntR1* (*gntR1-E70K*) and *ramA* (*ramA-A52V*) in the wild-type *C. glutamicum* background, resulting in a specific growth rate of 0.62 h^−1^ with an increased sugar consumption rate of around 30% in the minimal medium [304]. Therefore, better growth cannot be directly related to the increased concentration of products.

## 9. Conclusions

Phenolic acids have gained attention due to their potential health benefits, having already found applications in the agricultural, medicinal, cosmetic, nutraceutical, and food industries. From a human physiological standpoint, phenolic acids are reported to have anti-aging, anti-inflammatory, antioxidant, and anti-proliferative activities. Because of their pharmacological action and commercial value, the demand for phenolic acids is increasing. Naturally derived phenolic acids are produced as secondary metabolites by plants and engineered or natural microorganisms (bacteria, fungi, yeasts). Numerous studies, based on genetic or metabolic engineering, have focused on improving the production of phenolic acids. Additionally, metabolic engineering has enabled microbial cell factories to produce these high-value chemicals in a sustainable way. Agro-industrial wastes are also regarded as potential feedstocks for the recovery of phenolic acids. High phenolic acid yields can also be obtained in fermentation processes using natural and engineered microorganisms. The application of new genetic engineering tools enables the construction of the pathways in yeast and bacteria that produce the highest titers of phenolic acids. The optimisation of carbon flux and transcription factors, the overexpression of key genes in synthetic pathways in engineered microorganisms, and the application of the new tools of genetic engineering (CRISPR) allows us to obtain the highest titers (>10 g/L) of phenolic acids (*p*-hydroxybenzoic, salicylic, *p*-coumaric acids) using cheap carbon sources such as glucose. For other phenolic acids, biosynthesis is still challenging, and the product titers should be at least 10-fold higher for economically feasible production. In some cases (e.g., for ferulic or vanilic acids), enzymatic conversion or bio-refining can be a more promising approach. For some phenolic acids, especially gentisic, syringic, sinapic, and caffeic acids, plants remain as the best source. Production of phenolic acids such as isovanillic, everninic, *o*- and *m*-coumaric acids, and β-resorcylic acids is still not possible by microbial cell factories. Although phenolic acids such as *p*-hydroxybenzoic acid, *p*-coumaric acid, caffeic acid, vanillic acid, gallic acid, syringic acid, and ferulic acid can be used directly in various applications, their value can be significantly increased when they are further modified to high value-added compounds [305]. During the past few years, the production of value-added compounds from natural sources has gained tremendous importance.

Besides classic analytical techniques, biosensors can also be applied for quantitative analysis of phenolic acids. Nowadays, the use of TF-based biosensors is also an attractive method; however, such sensors are restricted to certain phenolic acids, which shows the need for TF based on other phenolic acids. Among the emerging production techniques, biotechnology-based generation using fermentation of genetically engineered microorganisms shows great potential as an alternative to the current manufacturing systems and has actually been applied for the industrial supply of bioactive compounds. There is a strong future drive to screen and engineer microbial strains for phenolic acid production through improving carbon-flux, enzymes bioactivity, and creating synthetic pathways for phenolic acid biosynthesis. These tasks are of great importance, and they can be solved by employing multiple disciplines such as chemistry, biology, process engineering, and computational biology.

## Figures and Tables

**Table 1:** The structure of hydroxybenzoic acids. The table represents the functional groups of aromatic ring.

	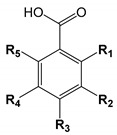					
**Number**	**Hydroxybenzoic Acid**	**R_1_**	**R_2_**	**R_3_**	**R_4_**	**R_5_**
**1**	*p*-Hydroxybenzoic acid			OH		
**2**	Salicylic acid	OH				
**3**	*m*-Hydroxybenzoic acid		OH			
**4**	Vanillic acid		CH_3_O	OH		
**5**	Isovanillic acid		OH	CH_3_O		
**6**	5-Hydroxyisovanillic acid		OH	CH_3_O	OH	
**7**	*o*-Vanillic acid	OH	CH_3_O			
**8**	Gallic acid		OH	OH	OH	
**9**	Hypogallic acid	OH	OH			
**10**	Protocatechuic acid		OH	OH		
**11**	Syringic acid		CH_3_O	OH	CH_3_O	
**12**	Gentisic acid	OH			OH	
**13**	*α*-Resorcylic acid		OH		OH	
**14**	*β*-Resorcylic acid	OH		OH		
**15**	*γ*-Resorcylic acid	OH				OH
**16**	Orsellinic acid	OH		OH		CH_3_
**17**	*p*-Orsellinic acid	OH		CH_3_		OH
**18**	*o*,*o*-Dimethylorsellinic acid	CH_3_O		CH_3_O		CH_3_
**19**	3-Methylsalicylic acid	OH	CH_3_			
**20**	4-Methylsalicylic acid	OH		CH_3_		
**21**	6-Methylsalicylic acid	OH				CH_3_
**22**	Everninic acid	OH		CH_3_O		CH_3_
**23**	Olivetolic acid	OH		OH		C_5_H_11_

**Table 2:** The structure of hydroxycinnamic acids. The table represents the functional groups of aromatic ring.

	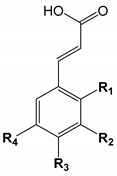				
**Number**	**Hydroxycinnamic Acids**	**R_1_**	**R_2_**	**R_3_**	**R_4_**
**24**	*o*-Coumaric acid	OH			
**25**	*m*-Coumaric acid		OH		
**26**	*p*-Coumaric acid			OH	
**27**	Ferulic acid		CH_3_O	OH	
**28**	5-Hydroxyferulic acid		CH_3_O	OH	OH
**29**	Isoferulic acid		OH	CH_3_O	
**30**	Sinapic acid		CH_3_O	OH	CH_3_O
**31**	Caffeic acid		OH	OH	
**32**	Umbellic acid	OH		OH	

**Table 3:** Yields of free phenolic acids extracted from plants.

Phenolic Acid	Plant Type and Part	Extraction Method	Solvent	Hydrolysis Applied *^a^	Yield (μg/g Dry Weight)	Reference
*p*-Hydroxybenzoic acid (**1**)	Maya nut (*Brosimum. alicastrum*)	Ultrasound-assisted extraction	Methanol–acetic acid	Alkaline	326.2	[95]
	Pulp from *Byrsonima ligustrifolia* fruits	Ultrasound-assisted extraction	Methanol, acetone	Alkaline, acidic	1000–2820	[96]
	Raspberry (*Rubus idaeus* L.) fruits	Percolation	Water–ethyl acetate	Acidic, alkaline	709.62	[32]
	Strawberry (*Fragaria ananassa*) fruits	Ultrasound-assisted extraction	Methanol with butylated hydroxyanisole −10% acetic acid	Acidic	44–63 ^b^	[97]
*m*-Hydroxybenzoic acid (**3**)	Melon (*Cucumis melo* L.) peels	Maceration	Ethanol–water	-	334.5	[98]
	Mangosteen (*Garcinia mangostana* L.) fruit peel	Maceration	Water–methanol	-	0.07	[99]
Salicylic acid (**2**)	Wheat (*Triticum aestivum* L.) straws	Maceration	Water	-	190.1	[100]
	Fresh red quinoa (*Chenopodium quinoa*) leaves	Accelerated solvent extraction	Methanol–water	-	0.48	[92]
Vanillic acid (**4**)	Blueberry (*Vaccinium myrtillus* L.) leaves	Ultrasound-assisted extraction	Methanol–formic acid, acetone–formic acid	-	1156.80	[88]
	Maya nut (*Brosimum. alicastrum*)	Sonication	Methanol–acetic acid	Alkaline	103	[95]
	Grape (*Citrus paradisi*) pomace	Ultrasound-assisted extraction	Ethanol–water	-	86	[101]
Isovanillic acid (**5**)	Melon (*C. melo* L.) peels	Maceration	Ethanol–water	-	237	[98]
Gallic acid (**8**)	Microalgae (*Ophiocordyceps sinensis)*	Maceration	Methanol	-	9489	[102]
	Clove (*Eugenia caryophylata *Thunb.) leaves	Maceration	Methanol–water	-	7385	[103]
	Black tea (*Clonorchis sinensis*) leaves	Percolation	Ethanol–water		6550	[104]
	Chinese olive *(Canarium album)* peel	Maceration	Methanol–acetic acid–water mixture		3696 ^b^	[105]
	Pulp from *B. ligustrifolia* fruits	Ultrasound-assisted extraction	Methanol, acetone, acetic acid	Acidic, alkaline	1485-30603	[96]
	Potatoes (*Solanum tuberosum* L.) peels	Maceration, ultrasound-assisted extraction	Ethanol–acetic acid	Acidic	2330	[106]
	Raspberry (*R. idaeus* L.) fruits	Maceration	Water	Acidic, alkaline	1669	[32]
Protocatechuic acid (**10**)	Chia (*Salvia hispanica* L.) seeds	Maceration	Methanol–water	-	759	[107]
	Cocao (*Theobroma cacao*) powder	Maceration	Methanol, acetic acid, butylated hydroxyanisole	Acidic, alkaline	400	[97]
	Red onion (*Allium cepa* L.) outer layer	Maceration	Methanol–water	-	354	[108]
	Araticum (*Annona crassiflora Mart*.) fruit peels	Ultrasound-assisted extraction	Methanol–acetone–water	-	318	[109]
	Hyssop (*Hyssopus officinalis* L.) herb	Maceration	Methanol	-	310	[110]
	Star anise (*Illicium verum*) fruits	Maceration	Ethanol	-	209.7	[111]
Syringic acid (**11**)	Blueberry (*V. myrtillus* L.) fruits	Ultrasound-assisted extraction	Methanol–formic acid, acetone–formic acid	Acidic	5627.47	[88]
	Cashew (*Anacardium occidentale* L.) nut testa (defatted)	Maceration	Ethanol–water	Acidic, alkaline	2507	[112]
	Lemon balm (*Melissa officinalis* L.) plants	Percolation	Methanol–water	-	540.8	[113]
	Raspberry (*R. idaeus* L.) fruits	Percolation	Water–ethyl acetate	Acidic, alkaline	113.41	[32]
Gentisic acid (**12**)	Lavender (*Lavandula officinalis* L.) flowers	Maceration	Methanol	-	8600	[110]
	Bitter melon (*Momordica charantia* L.) fruits	Accelerated solvent extraction	Water	-	5910	[114]
	Strawberry (*Fragaria ananassa* L.) fruits	Percolation	Water–ethyl acetate	Acidic, alkaline	120	[32]
4-Methylsalicylic acid (**20**)	Blueberry (*V. myrtillus* L.) fruits	Maceration	Sodium carbonate solution	Acidic	24	[115]
	Beans (*Vicia faba* L.*)*	Maceration	Sodium carbonate solution	Acidic	0.92	[115]
3-Methylsalicylic acid (**19**)	Beans (*V. faba* L.*)*	Maceration	Sodium carbonate solution	Acidic	4.37	[115]
	Blueberry (*V. myrtillus* L.) fruits	Maceration	Sodium carbonate solution	Acidic	0.8	[115]
*p*-Coumaric acid (**26**)	Chokeberry (*Aronia melonocarpa* L.) fruits	Ultrasound-assisted extraction	Acidic water–ethanol	-	4020 ^b^	[116]
	Pulp from *B. ligustrifolia* fruits	Ultrasound-assisted extraction	Methanol, acetone, acetic acid	Alkaline, acidic	2898–5265	[96]
	Leaves of walnut tree (*Juglans regia* L.)	Ultrasound-assisted extraction	Methanol	-	1250	[117]
	Strawberry (*Fragaria ananassa* L.) fruits	Maceration	Water	Acidic, alkaline	1108	[32]
*o*-Coumaric acid (**24**)	Leaves of rubber vine (*Conradina grandiflora*)	Maceration	Methanol	-	45.55	[118]
	Leaves of moringa (*Moringa oleifera* L.)	Maceration	Methanol, ethanol, ethyl acetate, water, and acetone	-	37	[119]
	Barley (*Hordeum vulgare* L.) straws	Maceration	Water	-	1.8	[100]
*m*- Coumaric acid (**25**)	Rice (*Labelle*) hull	Maceration	Methanol–water	-	432	[120]
	Melon (*C. melo* L.) peels	Maceration	Ethanol–water	-	199.1	[98]
	Barley (*Hordeum vulgare* L.) straws	Maceration	Water	-	3.1	[100]
	Leaves of rubber vine (*C. grandiflora*)	Maceration	Methanol	-	1.02	[118]
Caffeic acid (**31**)	Roasted coffee beans ^c^	Maceration	Hot water	-	17,400	[97]
	Leaves of tansy (*Tanacetum vulgare* L.)	Ultrasound-assisted extraction	Methanol	-	8940	[117]
	Potatoes (*S. tuberosum* L.) peels	Maceration, ultrasound-assisted extraction	Ethanol–acetic acid	Acidic	3320	[106]
	Basil (*Ocimum basilicum* L.)herb	Maceration	Methanol	-	2600	[110]
	Mate (*Ilex paraguariensis* St. Hil.) leaves	Percolation	Ethanol–water	-	760	[104]
	Blackcurrant (*Ribes nigrum* L.) fruits	Maceration	Water	Acidic, alkaline	537	[32]
Ferulic acid (**27**)	Wheat (*T. aestivum* L.) aleurone	Maceration	Sodium hydroxide solution in water	Alkaline	>7000	[121]
	Wheat (*T. aestivum* L.) bran	Maceration	Methanol–water	-	2020	[122]
	Mate (*I. paraguariensis* St.Hil.) leaves	Percolation	Ethanol–water	-	1360	[104]
	Hyssop (*Hyssopus officinalis* L.) herbs	Maceration	Methanol	-	460	[110]
	Baru (*Dipteryx alata Vog.*) nuts	Maceration	Methanol–hydrochloric acid solution	-	454	[123]
	White onion (*A. cepa* L.) outer layer	Maceration	Methanol–water	-	116	[108]
Sinapic acid (**30**)	Mate (*I. paraguariensis*) leaves	Percolation	Ethanol–water	-	1870	[104]
	Defatted canola (*B*rassica *napus* L.) seeds	Ultrasound-assisted extraction	Methanol–water	-	590 ^b^	[94]
	Strawberry (*F. ananassa* L.) fruits	Maceration	Water	Acidic	445	[32]

*^a^—hydrolysis applied results in the increased concentration of free phenolic acids; *^b^*—on fresh matter; *^c^*—recalculated from coffee brew to coffee beans.

**Table 4:** Phenolic acid distribution in various species of mushrooms.

Phenolic Acid	Mushroom	Extraction Method	Solvent	Yield (μg/g DW)	Reference
Protocatechuic acid (**10**)	*Ramaria botrytis*	Maceration	Methanol	342.7	[156]
	*Morchella esculent*	Ultrasound-assisted extraction	Methanol	17.15	[41]
	*Ganoderma lucidum*	Ultrasound-assisted extraction	Methanol	3.01	[41]
*p*-Hydroxybenzoic acid (**1**)	*Agaricus brasiliensis*	Maceration	Ethanol–water	332.76	[34]
	*Agaricus silvicola*	Maceration	Methanol	238.7	[156]
	*R. botrytis*	Maceration	Methanol	14.00	[156]
	*G. lucidum*	Ultrasound-assisted extraction	Methanol	5.22	[41]
Gallic acid (**8**)	*A. brasiliensis*	Maceration	Ethanol–water	491.89	[34]
	*M. esculent*	Ultrasound-assisted extraction	Methanol	0.7818	[41]
	*Rugiboletus extremiorientalis*	Ultrasound-assisted extraction	Water	0.03654	[38]
*p*-Coumaric acid (**26**)	*A. silvicola*	Maceration	Methanol	45.72	[156]
	*A. brasiliensis*	Maceration	Ethanol–water	24.47	[34]
	*Agaricus bisporus (white)*	Ultrasound-assisted extraction	Methanol–water mixture	2.31	[162]
	*G. lucidum*	Ultrasound-assisted extraction	Methanol	1.39	[41]
Ferulic acid (**27**)	*A. brasiliensis*	Maceration	Ethanol–water	752.54	[34]
	*M. esculent*	Ultrasound-assisted extraction	Methanol	0.075	[41]
	*R. extremiorientalis*	Ultrasound-assisted extraction	Water	0.001	[38]
Vanillic acid (**4**)	*G. lucidum*	Ultrasound-assisted extraction	Methanol	15.96	[41]
	*R. extremiorientalis*	Ultrasound-assisted extraction	Water	0.0113	[38]
Syringic acid (**11**)	*G. lucidum*	Ultrasound-assisted extraction	Methanol	2.34	[41]
	*R. extremiorientalis*	Ultrasound-assisted extraction	Water	0.0016	[38]
Sinapic acid (**30**)	*R. extremiorientalis*	Ultrasound-assisted extraction	Water	0.0022	[38]
Gentisic acid (**12**)	*A. brasiliensis*	Maceration	Ethanol–water	27.73	[34]

**Table 5:** The production of phenolic acids produced through biotransformation or fermentation using non-modified microorganisms.

Product	Initial Concentration	Final Concentration or Yield	Raw Material or Substrate	Fungi/Bacteria	Reference
*p*-Hydroxybenzoic acid (**1**)	3.48 ± 0.10 μg/g	21.80 ± 1.5 μg/g	Hemicelluloses from kidney bean extract	*B. subtilis*	[177]
	6.2 ± 1.8 mg/g_dry weight_	22.3 mg/g_dry weigt_	Lignin from rice bran	*R. oryzae*	[178]
Salicylic acid (**2**)	0 g/L	≈15 g/L	Naphtalene (2%)	*P. aeruginosa*	[168]
	0 mg/L	27.3 mg/L (0.13%)	Sucrose (80 mM/L) from RM2 medium	*R. gelatinosus* RASN4	[169]
Gallic acid (**8**)	0 g/g of biomass accumulated	7.35 g/g of biomass accumulated	Tannic acid	*A. fischeri* MTCC 150	[175]
	0%	94.8%	Tannic acid	*R. oryzae* (RO IIT RB-13, NRRL 21498) and *A. foetidus* (GMRB013 MTCC 3557)	[179]
	n.d.	154.5 mg/g_dry weight_	Lignin from rice bran	*R. oryzae*	[178]
	13.2 μg/mL	160 μg/mL	Palm oil mill effluent *	*A. niger IBS-103ZA*	[180]
Orsellinic acid (**16**)	0 mg/g	33 mg/g	Dextrose from Sabouraud medium	*P. polonicum* C3	[174]
Protocatechuic acid (**10**)	8.7 ± 1.2 mg/g_dry weight_	13.6 mg/g_dry weight_	Lignin from rice bran	*R. oryzae*	[178]
Vanillic acid (**4**)	0 mg/L	250 mg/L	Ferulic acid, 4 mmol/L	*S. thermophile*	[173]
	0 mg/L	365 mg/L (36.5%)	Ferulic acid, 1 g/L	*H. elognata*	[171]
	0%	57.3%	Ferulic acid	*P. lactis* SAMS-2001	[170]
Syringic acid (**11**)	2.6 ± 0.6 mg/g_dry weight_	12.7 mg/g_dry weight_	Lignin from rice bran	*R. oryzae*	[178]
	0 mg/L	85 mg/L	Sinapic acid (5 mM) solution in minimal medium	*P. variotii*	[167]
*p*-Coumaric acid (**26**)	71.8 μg/mL	146 μg/mL	Palm oil mill effluent *	*A. niger* IBS-103ZA	[180]
Caffeic acid (**31**)	1.6 ± 0.2 mg/g_dry weight_	28.7 mg/g_dry weight_	Lignin from rice bran	*R. oryzae*	[178]
	286 μg/mL	340 μg/mL	Palm oil mill effluent *	*A. niger* IBS-103ZA	[180]
Ferulic acid (**27**)	159 μg/mL	225 μg/mL	Palm oil mill effluent *	*A. niger* IBS-103ZA	[180]
	10.56 ± 2.46	69.98 ± 13.75 μg/g	Hemicelluloses from kidney bean extract	*B. subtilis*	[177]
	0%	85% of alcaline extracted compounds	Pectin in sugar beet pulp	*P. chrysogenum* 31B	[181]
Hypogallic acid (**9**)	0 mM	2.9 mM (50.4% yield)	*m*-Hydroxybenzoate	*P. testosteroni*	[182]

* The substrate is not designated.

**Table 6:** Transcription factor-based microbial biosensors for phenolic acids.

Target Molecule of Phenolic Acid	Sensing Element ^a^	Output Element ^b^	Dynamic Range (At the Concentration of Analyte)	Reference
Protocatechuic acid (**10**)	PcaU	Engineered P*_pcaU_*	14 (20 mM)	[287]
	PcaU^AM^	Engineered P*_pcaU_*, P*_3B5B_*	1.5; 1.8 (1 mM)	[285]
	PcaV	Engineered P*_Pv_*	3 (1 mM)	[288]
	PcaU	Engineered P*_pcaU_*	≈12 (0.003mM)	[289]
	Engineered PobR	P*_pobR_*	64 (10 mM)	[290]
Vanillic acid (**4**)	VanR	P*_TEF1_*	≈8 (4 mM)	[279]
	EmrR	P*_EmrR_*	1 (50 μM)	[291]
	Engineered EmrR	Engineered P*_EmrR_*,	9.5 (5 mM)	[292]
		P*_vtac_*,	6.8 (5 mM)	
		P*_vtrc_* and P*_vtic_*	2.1 (5 mM)	
	VanR-VanO	Engineered P*_VanAB_*	14 (1mM)	[293]
	VanR_am_	P*_VanCC_*	2.3 (100 μM)	[285]
*p*-Hydroxybenzoic acid (**1**)	EmrR	P*_EmrR_*	0.5 (1 mM)	[291]
	PobR variant	P*_pob_*	64 (1 mM)	[290]
	PobR variant	P*_pob_*	≈12 (30 mM)	[294]
	PcaV	Engineered P_Pv_	3.6 (1 mM)	[288]
*m*-Hydroxybenzoic acid (**3**)	PcaV	Engineered P_Pv_	2.8 (1 mM)	[288]
Salicylic acid (**2**)	AraC-TAL	P*_BAD_*	218 (5 mM)	[283]
	NahR^AM^	P*_salTCC_*	2.1 (100 μM)	[285]
	Engineered AraC	P*_BAD_*	≈200 (5 mM)	[284]
	SalR	P*_sal_*	10 uM	[295]
4-Methylsalicylic acid (**20**)	NahR, NahF-R	P*_J23114_*	≈2 (1 mM)	[296]
3-Methylsalicylic acid (**19**)	XylS	P*_m_*	≈2 (1 mM)	[296]
	NahF-R	P*_J23114_*	≈2 (1 mM)	
Hypogallic acid (**9**)	NahR, NahF-R	P*_J23114_*	≈2 (1 mM)	[296]
*p*-Coumaric acid (**26**)	EmrR	Engineered P*_EmrR_*: *P_vtac_*, *P_vtrc_*	10.4 and 8.5 (1 mM)	[292]
	AraC-TAL	P*_BAD_*	2.3 (5 mM)	[283]
	FerC	Engineered P*_LC_*	25 (1 mM)	[286]
	PadR	P*_padC_*	≈130 (2 mM)	[282]
Ferulic acid (**27**)	FerC	Engineered P*_LC_*	26.2 (1 mM)	[286]
Caffeic acid (**31**)	FerC	Engineered P*_LC_*	11.2 (1 mM)	[286]
Sinapic acid (**30**)	FerC	Engineered P*_LC_*	15.4 (1 mM)	[286]
Umbellic acid (**32**)	FerC	Engineered P*_LC_*	9.6 (1 mM)	[286]
5-Hydroxyferulic acid (**28**)	FerC	Engineered P*_LC_*	14.8 (1 mM)	[286]
Isoferullic acid (**29**)	FerC	Engineered P*_LC_*	33.5 (1 mM)	[286]

^a^ Transcription factors involved in controlling genes encoding phenolic or aromatic compounds metabolic pathways; ^b^ promoters controlled by relevant transcription factor.

## Supplementary Materials

The following are available online at https://www.mdpi.com/2218-273X/10/6/874/s1, Table S1: Phenolic acid production in genetically engineered bacteria, yeasts, and fungi.
