# Supplementary material for: Advances and Prospects of Phenolic Acids Production, Biorefinery and Analysis
Source: Biomolecules. 2020 Jun 6;10(6):874. doi: 10.3390/biom10060874 (PMC7356249; doi:10.3390/biom10060874)
Supplement: Supplementary file 1 [file biomolecules-10-00874-s001.pdf]

Supplementary Materials

Advances and prospects of phenolic acids production, biorefinery and analysis

Egle Valanciene[a], Ilona Jonuskiene[a], Michail Syrpas[a], Ernesta Augustiniene[a], Paulius Matulis[a], Andrius Simonavicius[a], Naglis Malys[a]*

[a]Bioprocess Research Centre, Faculty of Chemical Technology, Kaunas University of Technology, Radvilėnų pl. 19, Kaunas, Lithuania

*Corresponding author, email: naglis.malys@ktu.lt

Table S1. Phenolic acid production in genetically engineered bacteria, yeasts and fungi

Phenolic acid (product)	Microorganism	Genes			Carbon source (concentration)	Precursor	Fermentation conditions	Titre	Yield	Reference[b]
		Inserted	Overexpressed	Deleted						
p-Hydroxybenzoic acid (**1**)	*S. cerevisiae*	*aro3, aro4*	*aro4*^{K229L}, *aroL, ubiC*	*aro7, trp3*	Glucose (20 g/L)	Not added	Fed batch 5 mmol/(g_{CDW} × h)	2.9 g/L	3.1 mg/g glucose	[234]
	C. glutamicum		*tal, tkt, aroG, aroB, aroD, aroE, aroK, aroC, aroA, ubiC*	*ldhA, qsuB, qsuD, pyk, hdpA, ldhA*	Glucose (consumed 638.5 mmol)	Not added	batch	36.6 g/l	41 mol/mol glucose	[199]
	C. glutamicum APS809	*aroA*	*aroG*^{fbr}, *aroF*^{fbr}, *aroB, qsuB, aroK, aroC, ubiC*	*qsuB, qsuD, trpE, pobA, csm*	Glucose (80 g/l)	Not added	Fed batch, 7x100 mL solution (498 g glucose)	137.6 mM (19 g/l)	9.65 %	[235]
	P. taiwanensis		*tktA, pgi, ppsA, aroG*^{fbr}, *tyrA*^{fbr}, *pal/tal, fcs, ech, vdh*	*quiC, quiC1, quiC2, hpd, trpE, pobA*	Glycerol (40 mM)	Not added	Shake flask	5.1 mM	29.6 % (mol/mol)	[236]
m-Hydroxybenzoic acid (**3**)	*C. glutamicum*	*iolT1, aroF, qsuB, hyg5*	*tkt*	*phdBCDE* *qsuB, pobA,* *pcaFDOCBGHI* *catCBA, benABCD,*	Glucose (4%, 222 mM)	Not added	Shake flasks	0.30 g/L (2.2 mM)	NR[a]	[218]

				nagLKIRT, genH						
Salicylic acid (**2**)	*C. glutamicum*	*iolT1-, aroF, qsuB, irp9*	*tkt*	*phdB,C,D,E*<br>*qsuB, pobA,*<br>*pcaF,D,O,C,B, G,H,I*<br>*catC,B,A, benA,B,C,D, nagL,K,I,R,T, genH*	Glucose (4%; 222 mM)	Not added	Shake fasks	10 mg/L (0.07 mM)	NR	[218]
	E. coli	*galP/glk, ppsA, ppc, menF, pchB*		*pykF, pykA, pheA, tyrA*	Glucose (20 g/l)	Not added	Batch	11.5 g/L	40 % (mol/mol)	[237]
Vanillic (**4**)	*P. fluorescens* BF13-97	-	-	*vanAB*	*p*-Coumaric acid<br>(0.1% wt/vol)	*p*-Coumaric acid	Shake flask	~0.29 mg/ml	NR	[205]
	P. putida KT2440	*vanAB (AD^{-})*	-	-	Ferulic acid	Ferulic acid (~1.7 g/l)	Shake flasks	~1.6 g/l	95 % (mol/mol)	[224]
					Ferulic acid	Ferulic acid extract of corn bran (~1.5 g/l)		~1.15 g/l	87 % (mol/mol)	
Protocatechuic acid (**10**)	*C. glutamicum*	*iolT1^{-}, aroH, qsuB, qsuB*	*tkt*	*phdB,C,D,E*<br>*qsuB, pobA,*	Glucose (222 mM)	Not added	Shake flasks	2.0 g/L (13.0 mM)	NR	[218]

				pcaFDOC,B,G, H,I<br><br>*catC,B,A, benA,B,C,D, nagL,K,I,R,T, genH*						
	S. cerevisiae	-	*aroZ, aroB, aroD*	*pheA, aroE*	Glucose (20g/l)	Not added	Shake flasks	0.15 g/l	NR	[238]
	E. coli	*aroB, aroZ,*	*tktA, DAHP*^{fdi}	*serA*	Glucose (30 g/l)	*p*-Hydroxybenzoic acid (0.01 g)	Shake flasks	41 g/l	26 % (mol/mol)	[239]
Gallic acid (**8**)	*E. coli*	*pobA*, ubiC*	*aroL, ppsA, tktA, aroG*^{fbr}	-	Glycerol (10 g), glucose (2.5 g)	*p*-Hydroxybenzoic acid (1 g/l)	Shake flasks	1266.39 mg /L	NR	[240]
Gallic acid (as by product) (**8**)	*E. coli*	*aroB, ppsA*<br><br>*aroF*^{fbr}<br><br>*aroB,D*	*tktA*	*aroE*	Glucose (30 g/L)	3-Dehydroshikimic acid	Fed-batch,<br><br>(10-30 % of glucose maintained)	13 g/l	NR	[206]
Hypogallic acid (**9**)	*E. coli*	*aroL, ppsA, tktA, aroG*^{fbr}	*entCBA*	*EntE*	Glycerol (10 g/l), glucose (2.5 g/l)	Not added	Shake flask	900.03 mg/l	NR	[221]
Gentisic acid (**12**)	*E. coli*	*galP, glp, hyg5, aroF, aroG, cgl3026*	-	*pykF, pykA, pheA, tyrA, ptsH, ptsI*	Glucose (5.7 g/l)	Not added	Test-tube cultivation	1050 mg/L	NR	[241]

Syringic acid (**11**)	*E. coli*	*desV, ligV*	-	-	Syringaldehyde (5mM)	Syringaldehyde	Batch	NR	NR	[225]
α-Resorcylic acid (**13**)	*A. oryzae*	-	*csyA*	-	Maltose	Not added	Batch	5 mg/l	NR	[226]
6-Methylsalicylic acid (**21**)	*P. pastoris*	*npgA, atx*	-	-	Glycerol, methanol	Not added	Fed batch<br>glycero medium (50%)l: 2h x 8 mL/L/h;<br>methanol: 4 mL/h/L for 2 hours and encreased to 12 mL/h/L untill the end	2.2 g/l	NR	[227]
	E. coli	*Pg6MSAS-sfp*, anti-*pabA, sfp*	***accBCD1, serA***	-	Glycerol (100 g/l)	Not added	Fed-batch with 800g/l glycerol (when ph>6.86)	440.3 mg/l	NR	[231]
	K. phaffii	-	***HRK1, ScACS1****	-	Glucose (1%)	Acetate (20 mM)	Fed batch, acetate (2x20 mM), glucose (2x 0.5 %)	113.6 mg/L	NR	[228]
Olivetolic acid (**23**)	*E. coli*	-	*ACC, fadD, OLS, OAC,*<br>*BktB, FadB, FadB, egTER*	*ldhA, frdA, adhE, pta, poxB*	Glycerol (8.5 g/l)	Hexanoate (80 mg/l)	Batch	80 mg/l	NR	[233]

p-Coumaric acid (**26**)	*E. coli*	*TAL, C3H, CYP199A2, Pdr, Pux, 4CL1, DCS , CURS1*	-	-	Glucose (40g/l)	L-Tyrosine 3 mM)	Shake flask	2.5 mM; (421.69 mg/L)	NR	[204]
	S. cerevisiae	*EcaroLZmtyrC, GmPDH1, MtPDH1, AtPAL, AtC4, AtATR, CYB, FjTAL*	*aro3, aro4^{K229L,} aro1, aro2, aro7^{G141S}, pha2, aro8*	*aro10, pdc5, gal80*	Glucose (20g/l)	Not added	Fed-batch (continous, glucose 200g/l)	12.5 g/l	154.9 mg/ g glucose	[208]
	E. coli	*TAL tyrA^{fbr}, ppsA, tktA, aroG^{fbr}*	-	*pheA, tyrA*	Glucose (1g/l)	Yeast extract (1 g/l)	Shake flask	475.4 mg/l	NR	[242]
	S. cerevisiae	*pha2, aro3, aroH*	*aro4^{fbr}, aroF^{fbr}, aroG^{fbr}, aroB, aroD, aroE, ydiB, aroL, aroK, aroA, aroC, Aro2, aro7^{fbr}, tyr1, tyrA^{fbr}, TAL*	*aro10, pdc5*	Glucose (2%)	Not added	Single -fed-batch	1.93 g/l	NR	[243]
Caffeic acid (**31**)	*E. coli*	*tyrA^{fbr}, ppsA, tktA, aroG^{fbr} , hpaBC 4HPA3H*	-	*pheLA-tyrA*	Glycerol, 20g/l	*p*-Coumaric acid 3 g/l	Fed-Batch (2x05g/l)	3.82 g/l	NR	[244]

	S. cerevisiae	*hpaB*, *hpaC*, *PAL*, *TAL*, *C4H*	-	-	Glucose, 2g/l	Not added	Shake flask	289.4 ± 4.6 mg/l	NR	[214]
	E. coli F185L	*pux, pdr, CYP199A2*	-	-	Glycerol (10 % vol/vol)	*p*-Coumaric acid (20mmol)	Shake flask	2.8 g/l	NR	[210]
Ferulic acid (**27**)	*E. coli* B-FA1	*TAL, aroG, tyrA, Sam5, PAL*	-	*tyrR*	Glucose, 2%	Tyrosine (80 mg/L) from yeast extract	Shake flask	64 mg/L	NR	[209]
Sinapic acid (**30**)	*E. coli*	*ref1*	-	-	Glycerol (10% v/v)	Sinapaldehy de	Test-tube culture	NR	NR	[217]

[a] - not reported (NR);
[b] – all references are listed in the article.
